# FGF receptor inhibitor BGJ398 partially rescues osteoarthritis-like phenotype in older high molecular weight FGF2 transgenic mice via multiple mechanisms

**DOI:** 10.1038/s41598-022-20269-6

**Published:** 2022-09-24

**Authors:** Marja M. Hurley, J. Douglas Coffin, Thomas Doetschman, Christina Valera, Kai Clarke, Liping Xiao

**Affiliations:** 1 Department of Medicine, School of Medicine, UConn Health, 263 Farmington Avenue, Farmington, CT 06030-3023 USA; 2 Department BMED, SB 271, The University of Montana, Missoula, MT 59812 USA; 3 Department of Cellular and Molecular Medicine, University of Arizona College of Medicine, Tucson, AZ 85724 USA

**Keywords:** Metabolic bone disease, Osteoimmunology

## Abstract

We have used Basic Fibroblast Growth Factor (FGF2) transgenic mice as experimental models for human X-linked hypophosphatemia (XLH)-related degenerative osteoarthritis (OA) to investigate the pathogenesis of the disease and to test potential pharmacotherapies for treatment. This study tested the efficacy of BJG398, a small molecule fibroblast growth factor receptor tyrosine kinase (FGFRTK) inhibitor, to rescue the knee joint osteoarthritis phenotype in High Molecular Weight fibroblast growth factor 2 transgenic (HMWTgFGF2) mice. BJG398 was administered in vivo to 8-month-old female HMWTgFGF2 mice for six weeks. Histomorphometry, immunohistochemistry and micro-CT were used to examine the knee joints in BGJ398-treated and control mice. We assessed: Fibroblast Growth Factor 23 (FGF23) expression and FGFR1 activity; Matrix metalloproteinase 13 (MMP13) and Aggrecanase2 (ADAMTS5) expression; then signaling by SMAD1/5/8-pSMAD6, pERK1/2 and Runt-related transcription factor 2 (RUNX2). Using PrimePCR arrays, we identified a contributing role for major target genes in the TGFB/BMP2 signaling pathway that were regulated by BGJ398. BGJ398 inhibited HMWFGF2/FGF23-induced increase in bone morphogenic protein receptor-1, bone morphogenic protein-2 and 4 and Serine peptidase inhibitor, clade E, member 1. The results from Micro-CT and histology show BGJ398 treatment rescued the OA changes in subchondral bone and knee articular cartilage of HMWTgFGF2 mice. The gene expression and signal transduction results provide convincing evidence that HMWFGF2 generates OA through FGFRTK with characteristic downstream signaling that defines OA, namely: increased FGF23-FGFR1 activity with BMP-BMPR, activation of pSMAD1/5/8-RUNX2 and pERK signaling pathways, then upregulation of MMP13 and ADAMTS5 to degrade matrix. BGJ398 treatment effectively reversed these OA molecular phenotypes, providing further evidence that the OA generated by HMWFGF2 in the transgenic mice is FGFR-mediated and phenocopies the OA found in the Hyp mouse homolog of XLH with a spontaneous mutation in the Phex (phosphate regulating endopeptidase on the X chromosome) gene and human XLH-OA. Overall, the results obtained here explain how the pleotropic effects of FGF2 emanate from the different functions of HMW protein isoforms for cartilage and bone homeostasis, and the pathogenesis of XLH-degenerative osteoarthropathy. BGJ398 inhibits HMWFGF2-induced osteoarthritis via multiple mechanisms. These results provided important scientific evidence for the potential application of BGJ398 as a therapeutic agent for osteoarthritis in XLH.

## Introduction

Degenerative osteoarthropathy is a form of human osteoarthritis (OA) that is prevalent in young individuals with X-linked hypophosphatemia (XLH)^1^, and degenerative OA is common to all older XLH individuals where it is associated with significant morbidity^2,3^. Currently, there is no effective pharmacotherapy to treat XLH-OA. Hyp mice have a homologous phenotype to human XLH^2^ that also develops severe OA^4,5^. We previously reported that transgenic mice overexpressing the high molecular weight (HMW, nuclear isoforms) of FGF2 (HMWTgFGF2)^6^ demonstrate a rickets/osteomalacia phenotype. We then showed that HMWTgFGF2 mice develop knee joint OA as early as 2 months of age that progresses to complete joint destruction by 18 months of age^7^. The OA of HMWTgFGF2 mice is phenotypically similar to Hyp mice and to XLH human subjects^7^. HMWTgFGF2 mice have increased FGFR1 mRNA and protein expression in their knee joints^7^ and increased serum levels of FGF23. These results suggest a link between FGF23-related hypophosphatemia and the OA phenotype in HMWTgFGF2 mice.

FGF2 has been shown to regulate normal cartilage and joint homeostasis^8,9^. However, FGF2 also accumulates in OA synovial fluid where it simulates degenerative enzymes MMP-13, disintegrin and ADAMTS-5^8,10^. Moreover, germline deletion of the Fgf2 gene in mice, results in OA^10,11^. These seemingly conflicting results may be due to a prior lack of focus on distinct roles for the different FGF2 protein isoforms^12^. Hence, our studies using FGF2 isoform-specific transgenic mice have helped resolve these issues by demonstrating different roles for FGF2 protein isoforms in OA^11^.

Specifically, we have used FGF2 isoform-specific knockout mice^13^ (i.e. different lines that selectively lack either LMW or HMW FGF2 isoforms) to show that ablation of the low molecular weight 18 kDa isoform (LMWFGF2)^11^ or overexpression of the nuclear-localized high molecular weight isoforms (HMWFGF2)^6^ in mice results in OA^7^. Then, we showed that ablation of HMWFGF2 isoforms protects against OA development in mice^11^. Further support for this model, where LMWFGF2 and HMWFGF2 isoforms differentially regulate cartilage and joint homeostasis, may be evident in FGF2 isoform-mediated modulation of FGF2 Receptor Tyrosine Kinases and their downstream signaling pathways. Those receptors and their associated signaling pathways have been shown to either promote or protect against development of OA in humans and mice^14–20^.

Bone Morphogenic Proteins (BMP’s) function downstream from FGFRs, and similar to FGF2-FGFR function, published data show that members of the BMP family can be protective or contribute to OA in humans and mice^21^. BMP2 signaling has a key function in vertebrate cartilage development, stimulating both proliferation and anabolic gene expression^21^. It is also an important OA mediator, based on BMP2 ligand expression that is detected in human adult articular cartilage. BMP2 mRNA and protein expression is up-regulated in OA articular cartilage and OA articular cartilage-derived monolayer cell cultures^22–25^.

However, there are no published data showing how abnormal BMP2-BMP-receptor signaling contributes to the specific osteoarthropathy observed in HMWTgFGF2 mice, Hyp mice or humans with XLH. Our studies documented increased BMP mRNA in the knee joints of 2-month-old HMWTgFGF2 mice, compared to age-matched vector controls^7^ but we have not explored the potential contribution of BMP-BMPR activity and downstream signaling to the observed OA phenotype.

We previously reported that antagonism against FGFR prevented the development of OA in weanling HMWTgFGF2 mice^26^. This project was designed to examine whether an FDA approved FGFRTK inhibitor, BGJ398, could rescue fully developed, progressive OA phenotype in older HMWTgFGF2 mice; and to examine whether altered BMP signaling contributes to the observed catabolic changes. The pertinent, significant results reported here demonstrate that cross-talk between HMWFGF2 and BMP signaling contributes to OA in HMWTgFGF2 mice with a phenotype that is synonymous with OA in Hyp mice and humans with XLH. Furthermore, this study shows, for the first time, the efficacy of FGFR antagonism in well-developed murine XLH-OA that could translate to human OA pharmacotherapeutics.

## Results

### Knee X-rays and biomarker assays of 9.5-month-old vector-control and HMWTgFGF2 BGJ398-treated mice

**Figure 1:**
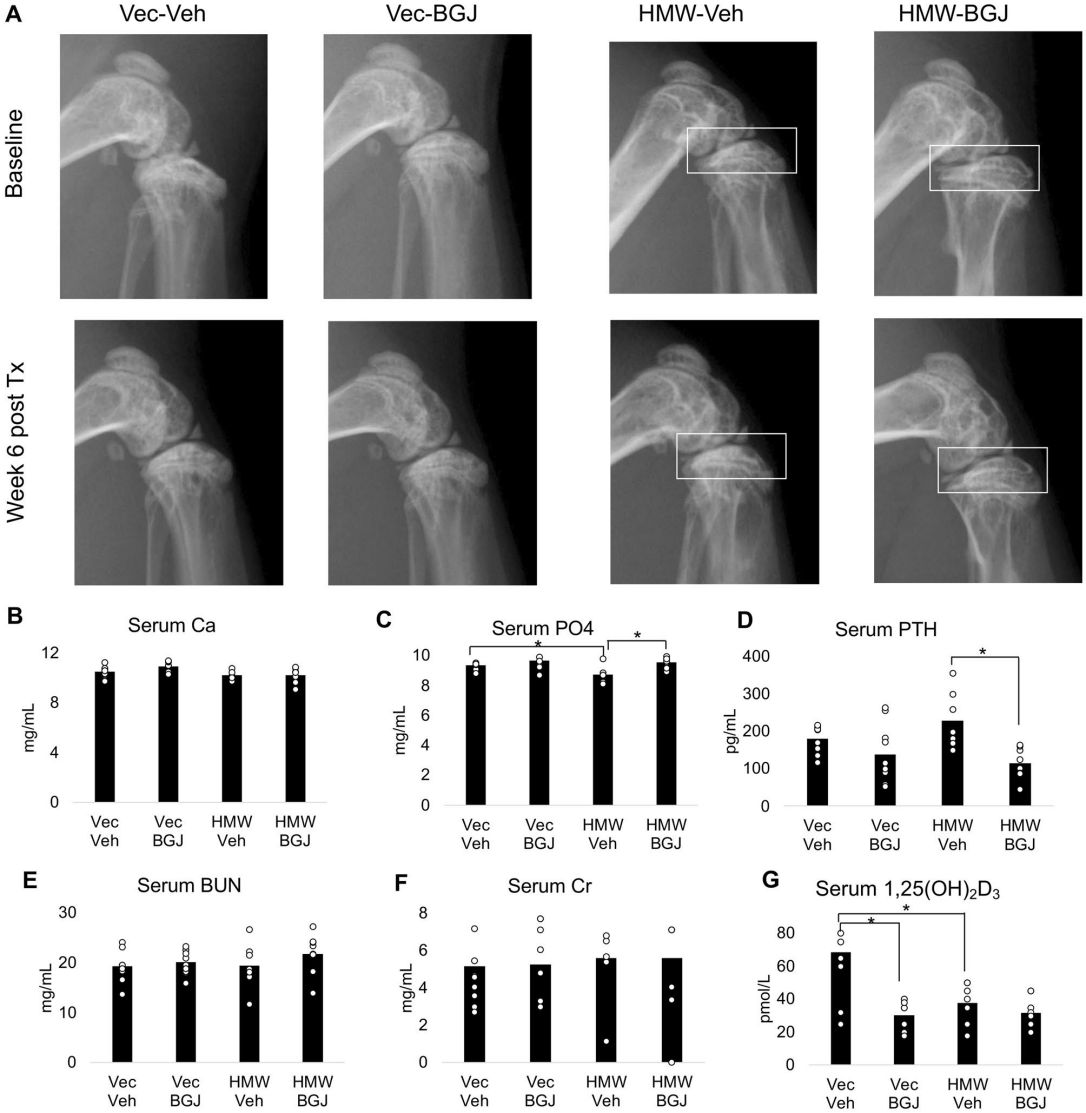
Knee X-rays and serum biomarkers of 9.5-month-old Vector-Control and HMWTgFGF2 mice treated with BGJ398. Eight-month-old Vector-Control (Vec) and HMWTgFGF2 (HMW) female mice were sq injected with vehicle or BGJ398 5 days/week for 6 weeks. (**A**) Representative digital X-ray image at baseline and at the end of treatment. X-rays showed the flattened tibia plateau and uneven joint surfaces in the HMWTgFGF2 mice before treatment, these were partially reversed by BGJ398 treatment but not with vehicle treatment (boxes). Representative images from n = 7–9 mice/group. Serum was collected during euthanasia at the end of experiment for serum biochemistry measurement (**B**–**G**). (**B**) Serum calcium, (**C**) phosphate, (**D**) parathyroid hormone, (**E**) blood urea nitrogen, (**F**) creatinine, (**G**) 1.25 dihydroxy vitamin D3. Data are shown as mean and individual points. n = 7–9 mice/group. *p < 0.05 by two-way ANOVA.

Our previous studies showed that FGFR inhibition prevented OA development in weanling HMWTgFGF2 mice^26^. Therefore, we assessed whether 6 weeks of in vivo BGJ398 treatment could partially or fully rescue severe knee OA in adult HMWTgFGF2 female mice that were 8 months old at the start of treatment and then sacrificed at 9.5 months. As shown in Fig. 1A, representative digital X-ray images (one at the start of treatment and another at the end) show that the flattened tibia plateau and uneven joint surfaces in the HMWTgFGF2 mice before treatment were partially reversed with BGJ398 treatment, but not with vehicle treatment. Similar results were obtained in 6.5-month-old HMWTgFGF2 male mice that were treated with BGJ398 for 6 weeks and sacrificed at 8 months of age as shown in the X-rays (Supplemental Fig. 1).

In order to assess whether BGJ398 affected biochemical markers of calcium, phosphate and renal homeostasis in older mice, and whether there were off-target side effects in Vector-Control mice, serum was collected at the termination of the experiment from both Vector-Control and HMWTgFGF2 mice treated with either vehicle or BGJ398. As shown in Fig. 1B, serum calcium was similar in all four groups. Figure 1C shows a significant reduction in serum phosphate in vehicle-treated HMWTgFGF2, that was reversed by BGJ398. Serum PTH (Fig. 1D) was significantly reduced by BGJ398 in HMWTgFGF2 mice. As shown in Fig. 1E, blood urea nitrogen (BUN) and creatinine (Fig. 1F) were similar in all four groups. Figure 1G shows serum 1,25 Dihydroxy Vitamin D3 (1,25(OH)_2_D_3_) was significantly reduced in vehicle-treated HMWTgFGF2 mice, but not increased by BGJ398; and BGJ398 significantly decreased 1,25(OH)_2_D_3_ in Vector-Control mice.

### Knee joint uCT shows BGJ398 rescue of abnormal structural parameters in epiphyseal subchondral HMWTgFGF2 femurs

**Figure 2:**
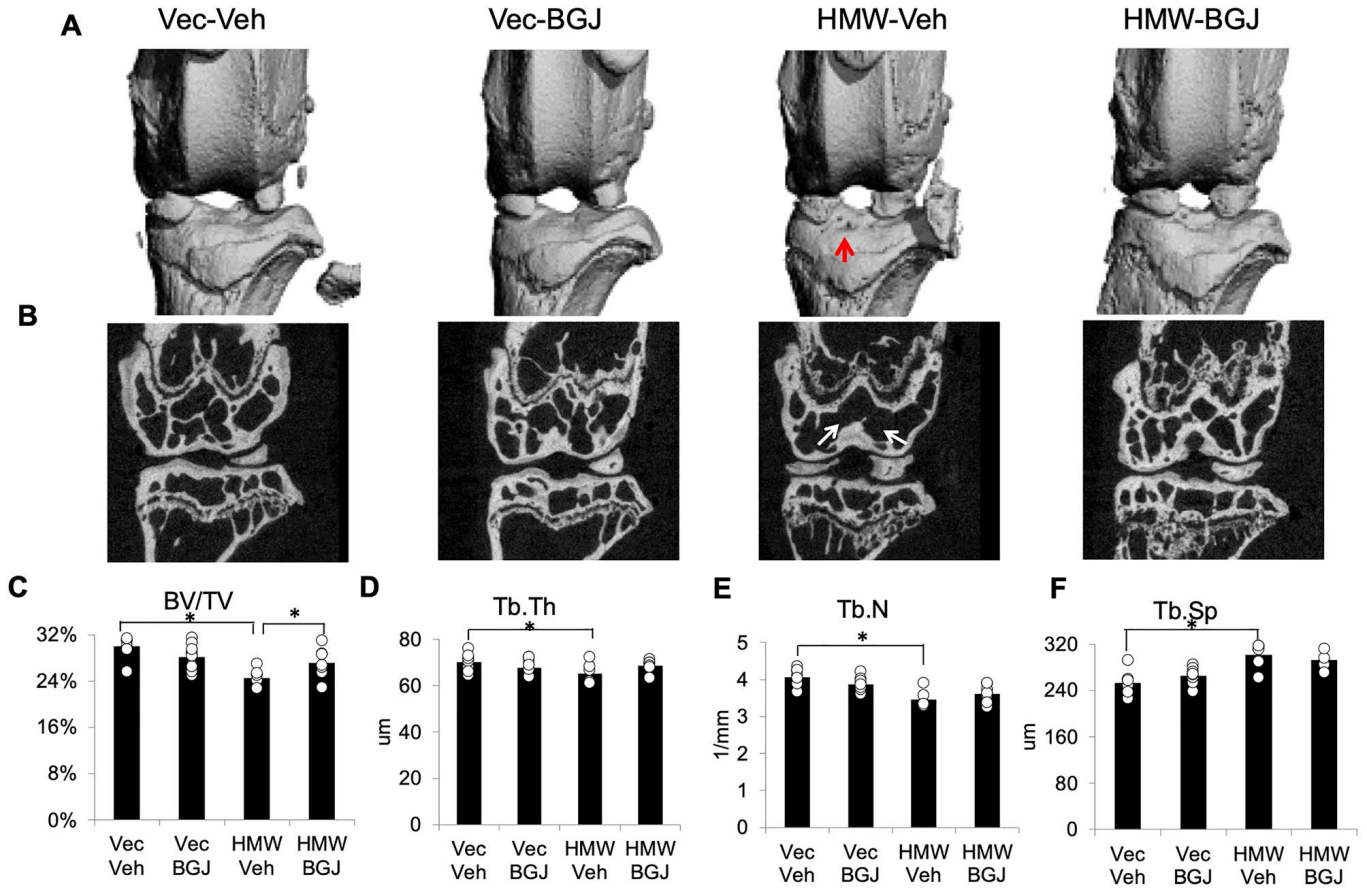
Micro-CT of knees from 9.5-month-old Vector-Control and HMWTgFGF2 mice treated with BGJ398. Eight-month-old Vector-Control (Vec) and HMWTgFGF2 (HMW) female mice were sq injected with vehicle or BGJ398 5 days/week for 6 weeks. Micro-CT analysis was performed at the end of experiment on excised knee joints. (**A**) Representative 3-dimensional Micro-CT images show erosion of articular surface in the HMWTgFGF2 vehicle treated group, that was partially rescued by BGJ398 treatment. (**B**) Representative 2-dimensional Micro-CT images reveal thinning and less subchondral bone in the HMWTgFGF2 vehicle-treated group that was rescued by BGJ398 treatment. Morphometric parameters calculated from 3-dimensional Micro-CT of femoral epiphyses of mice (**C**–**F**) showed significantly decreased bone volume per total volume (BV/TV), trabecular thickness (Tb.Th), trabecular number (Tb.N), and increased trabecular spacing (Tb.Sp) in femoral epiphysis of HMWTgFGF2 vehicle-treated knees compared with Vector-Control vehicle-treated epiphyses. BGJ398 treatment significantly increased abnormal BV/TV in HMWTgFGF2 mice. Data are shown as mean and individual points. n = 7–9 mice/group. *p < 0.05 by two-way ANOVA.

Micro-CT analysis was performed at the end of the experiment on excised knee joints from 9.5-month-old female (Fig. 2) and 8-month-old male (Supplemental Fig. 2) Vector-Control and HMWTgFGF2 female mice treated with either vehicle or BGJ398. As shown in Fig. 2A, erosion of articular surface in the HMWTgFGF2 vehicle-treated group was appreciated on gross examination of 3D reconstructions micro-CT images, that was partially rescued by BGJ398 treatment. Representative 2-dimensional Micro-CT images Fig. 2B, reveal thinning and less subchondral bone in the HMWTgFGF2 vehicle-treated group, that was rescued by BGJ398 treatment. Morphometric parameters calculated from 3-dimensional Micro-CT of femoral epiphyses (Fig. 2C) showed significantly decreased bone volume per total volume (BV/TV) in HMWTgFGF2 mice, that was significantly increased by BGJ398. However, reduced trabecular thickness (Tb.Th; Fig. 2D), trabecular number (Tb.N; Fig. 2E), and increased trabecular spacing (Tb.Sp; Fig. 2F) were all found in femoral epiphysis of HMWTgFGF2 vehicle-treated knees, compared with Vector-Control vehicle-treated epiphyses, without rescue by BGJ398 treatment. Histomorphometry analysis of the femoral subchondral bone from 9.5-month-old Vector-Control and HMWTgFGF2 mice treated with BGJ398 confirmed the micro-CT observation (Supplemental Fig. 3).

### BGJ398 rescues phenotypic osteoarthritis changes in knee articular cartilage of older HMWTgFGF2 female and male mice

**Figure 3:**
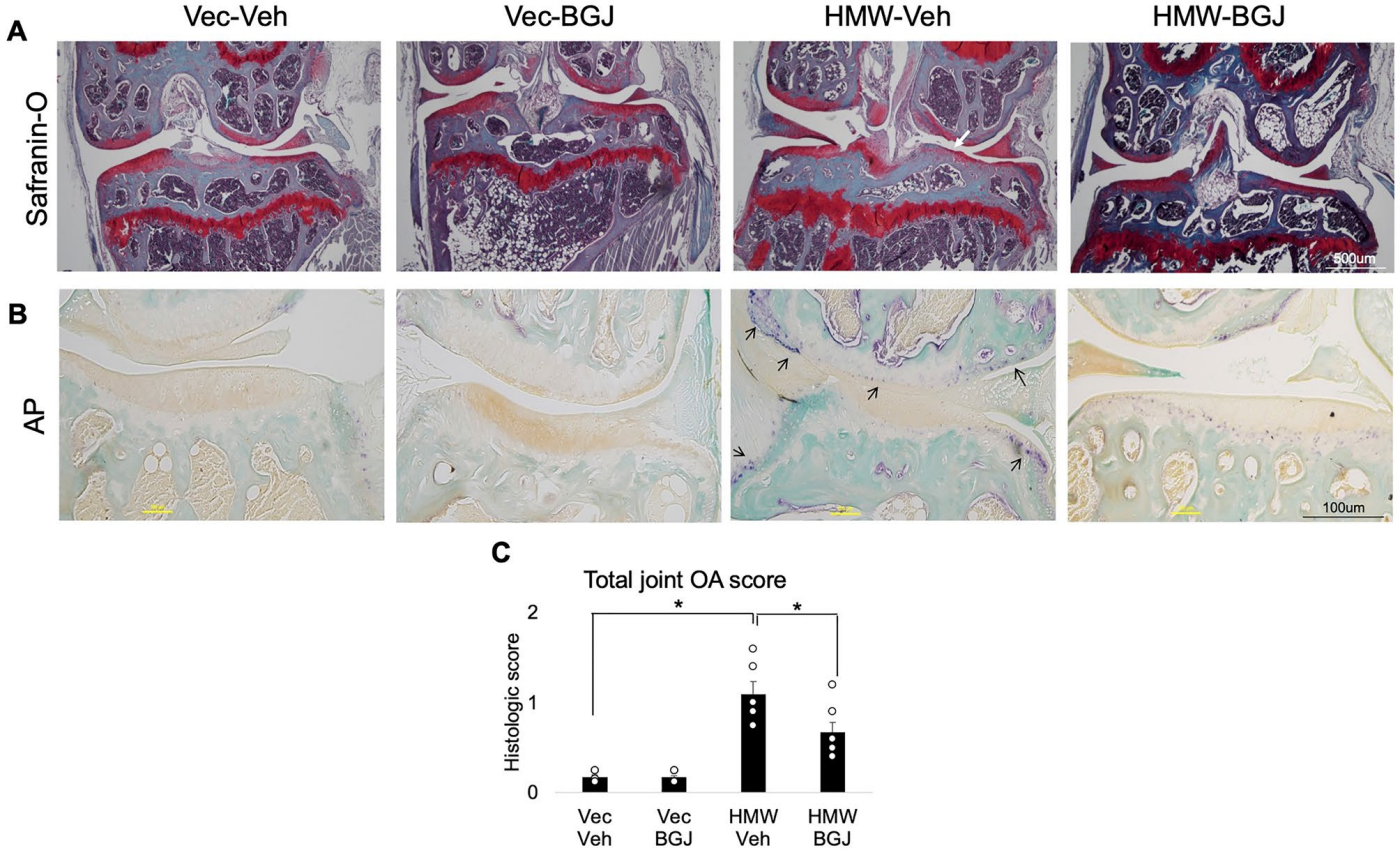
Histomorphometric analysis of knee articular cartilage of Vector-Control and HMWTgFGF2 mice treated with BGJ398. (**A**) Safranin-O-stained representative images of knee joint. Eight-month-old Vector-Control (Vec) and HMWtgFGF2 (HMW) female mice were sq injected with vehicle or BGJ398 5 days/week for 6 weeks. At the end of the experiment (9.5 months old), knee joints were collected for histology analysis. Safranin-O staining showed a dramatic decrease in proteoglycan content and erosion of cartilage surface in HMWTgFGF2 knees (white arrow), which was partially reversed by BGJ398 treatment. (**B**) Alkaline phosphatase staining of a representative knee joint. Six and a half-month-old Vector-Control and HMWTgFGF2 male mice were sq injected with vehicle or BGJ398 5 days/week for 6 weeks. At the end of experiment (8-month-old) knee joints were collected for histology analysis. Alkaline phosphatase staining showed more alkaline phosphatase-positive articular chondrocytes in HMWTgFGF2 vehicle-treated joints (black arrows), that was partially normalized by BGJ398 treatment. Representative images from n = 4 mice/group. (**C**) Mean total joint OA scoring was performed using Safranin-O-stained samples. OA score was significantly increased in the HWTg vehicle group compared with the Vector-Control vehicle group, that was partially rescued by BGJ398 treatment. Data are shown as mean and individual points. n = 6 mice/group. *p < 0.05 by two-way ANOVA.

At the end of the 6 week experiment, at 9.5 months of age, the knee joints of female Vector-Control and HMWTgFGF2 mice were collected for histology. As shown in Fig. 3A, compared with the Vector-Controls, Safranin-O staining showed a profound decrease in proteoglycan content and erosion of cartilage surface in HMWTgFGF2 knees (white arrow), and BGJ398 treatment partially rescued these phenotypic changes.

Histological analysis also defined the phenotype in the articular cartilage of knee joints of 8-month-old Vector-Control and HMWTgFGF2 male mice that were treated with either vehicle or BGJ398. Those treatments began at 6.6 months of age and were administered 5 days/week for 6 weeks. As shown in Fig. 3B, compared with the Vector-Control vehicle treated mice, there was a marked increase in alkaline phosphatase-positive articular chondrocytes in HMWTgFGF2 vehicle-treated joints (black arrows), that was partially reduced by BGJ398 treatment. As shown in Fig. 3C, mean total joint OA scoring (performed on Safranin-O stained samples) revealed an OA score that was significantly increased in the HMWTgFGF2 vehicle treatment group compared with the Vector-Control vehicle group; and, BGJ398 treatment significantly reduced that effect.

### Increased expression of matrix degrading enzymes in knee articular cartilage of 9.5-month-old HMWTgFGF2 mice is reversed by BGJ398

**Figure 4:**
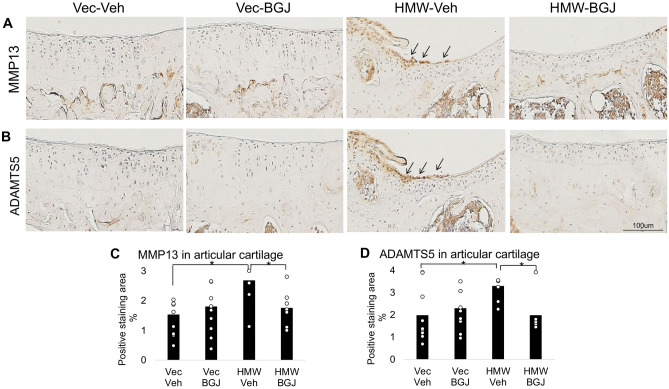
Expression of matrix degrading enzymes in knee articular cartilage of 9.5-month-old Vector-Control and HMWTgFGF2 mice treated with BGJ398. Eight-month-old Vector-Control (Vec) and HMWTgFGF2 (HMW) female mice were sq injected with vehicle or BGJ398 5 days/week for 6 weeks. At the end of the experiment (9.5-month- old), knee joints were collected for IHC staining. Representative immunohistochemical staining images for (**A**) MMP13 and (**B**) ADAMTS5. Quantitative analysis of percent-positive staining area for (**C**) MMP13 and (**D**) ADAMTS5 in articular cartilage. MMP13 and ADAMTS5 expression were increased in knees of HMWTgFGF2 vehicle-treated mice compared with Vector-Control vehicle-treated mice; these were rescued by BGJ398 treatment. Data are shown as mean and individual points. n = 7–9 mice/group. *p < 0.05 by two-way ANOVA.

We compared expression of the matrix degrading enzymes MMP13 and ADAMTS5 in 9.5-month-old Vector-Control and HMWTgFGF2 female mice that were treated with either vehicle or BGJ398 for 5 days/week, for 6 weeks. Knee articular cartilage immuno-stained for MMP13 (Fig. 4A) and ADAMTS5 (Fig. 4B) revealed increased expression for each enzyme in vehicle-treated HMWTgFGF2 mice, compared to the vehicle-treated Vector-Controls. Subsequent quantitative analysis of the percent-positive staining area for MMP13 (Fig. 4C) and ADAMTS5 (Fig. 4D) showed a significant increase in MMP13 and ADAMTS5 expression in knees of HMWTgFGF2 vehicle-treated mice, compared with Vector-Control vehicle-treated mice, that was significantly rescued by BGJ398 treatment.

### Expression of FGF2 and phospho-FGFR1 in knee joints of 9.5-month-old vector-control and HMWTgFGF2 mice treated with BGJ398

**Figure 5:**
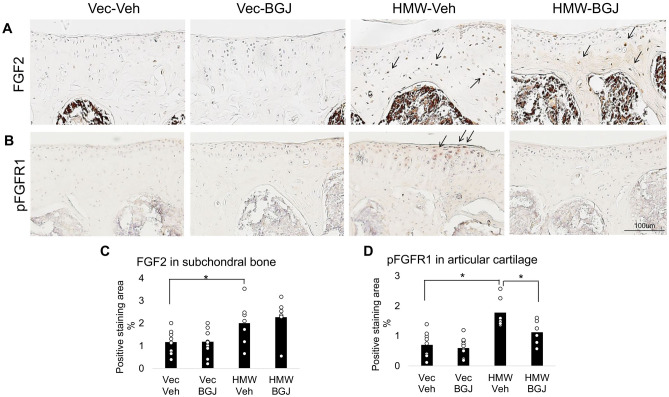
Expression of FGF2 in subchondral bone and phosphoFGFR1 in knee articular cartilage of 9.5-month-old Vector-Control and HMWTgFGF2 mice treated with BGJ398. Eight-month-old Vector-Control (Vec) and HMWTgFGF2 (HMW) female mice were sq injected with vehicle or BGJ398 5 days/week for 6 weeks. At the end of the experiment (9.5 months old), knee joints were collected for IHC staining. Representative immunohistochemical staining images for (**A**) FGF2 and (**B**) pFGFR1. Quantitative analysis of percentage of positive staining area for (**C**) FGF2 in subchondral bone and (**D**) pFGFR1 in articular cartilage. Data are shown as mean and individual points. n = 7–9 mice/group. *p < 0.05 by two-way ANOVA.

The localization and levels of expression of FGF2 and phosphoFGFR1 protein in the knee subchondral bone and articular cartilage of Vector-Control and HMWTgFGF2 female mice was assessed after vehicle or BGJ398 treatment for 6 weeks. Immuno-staining for FGF2 in subchondral bone (Fig. 5A) shows increased expression in HMWTgFGF2 vehicle-treated mice (relative to Vector-Control vehicle-treated mice) and BGJ398 treatment did not reduce the expression of FGF2 in HMWTgFGF2 subchondral bone. Increased expression of activated pFGFR1 (Fig. 5B) was observed in HMWTgFGF2 vehicle-treated knee articular cartilage relative to Vector-Control vehicle-treated mice. Quantitative analysis of the percent-positive staining area for FGF2 (Fig. 5C) showed no significant effect for BJG398 treatment in HMWTgFGF2 mice. However, as shown in Fig. 5D, BGJ398 treatment significantly reduced pFGFR1 in knee articular cartilage of HMWTgFGF2 mice. Although FGFR3 is reported to maintain articular chondrocytes in the undifferentiated stated^19^, we did not observe any significant differences in phosphoFGFR3 expression in the knee articular cartilage of vehicle or BGJ398-treated Vector-Control, or HMWTgFGF2 mice (Supplemental Fig. 4).

### Expression of FGF23 in knee articular cartilage, subchondral bone and serum of 9.5-month-old Vector-Control and HMWTgFGF2 mice treated with BGJ398

**Figure 6:**
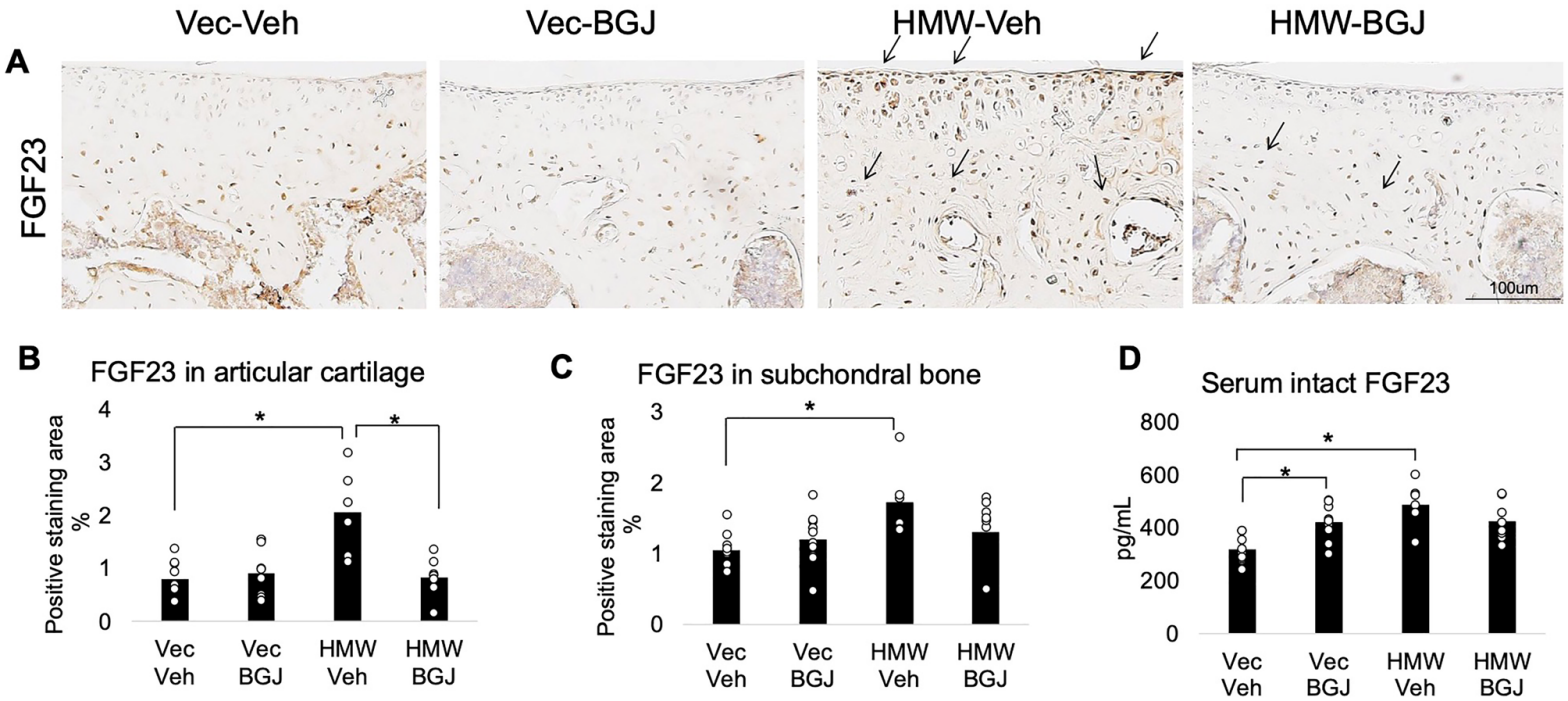
Expression of FGF23 in knee articular cartilage, subchondral bone and serum of 9.5-month-old Vector-Control and HMWTgFGF2 mice treated with BGJ398. Eight-month-old Vector-Control (Vec) and HMWTgFGF2 (HMW) female mice were sq injected with vehicle or BGJ398 5 days/week for 6 weeks. At the end of the experiment (9.5 months old), samples were collected. (**A**) Representative immunohistochemical staining images for FGF23. Quantitative analysis of percentage of positive staining area of FGF23 in (**B**) articular cartilage and (**C**) subchondral bone. (**D**) Serum intact FGF23 level was measured by ELISA. Data are shown as mean and individual points. n = 7–9 mice/group. *p < 0.05 by two-way ANOVA.

We previously reported that FGF23 was increased in bone osteocytes^6^, knee articular cartilage and serum from HMWTgFGF2 mice^7^. Therefore, we assessed the effects of BGJ398 treatment on FGF23 expression in all three of those tissues from Vector-Control and HMWTgFGF2 mice. Immunostaining for FGF23 (Fig. 6A) in articular cartilage and subchondral bone from vehicle-treated HMWTgFGF2 mice showed increased FGF23 (relative to Vector-Control vehicle treatment). BGJ398 treatment markedly reduced FGF23 staining in knee articular cartilage, but not subchondral bone in HMWTgFGF2 mice (relative to Vector-Control BGJ398 treatment). Subsequent quantitative analysis of the percent-positive staining area for FGF23 in articular cartilage (Fig. 6B) confirmed these results by showing that BGJ398 treatment significantly reduced FGF23 expression in HMWTgFGF2 mice. By contrast, BJG398 treatment did not significantly reduce increased FGF23 expression in subchondral bone (Fig. 6C). The serum level of intact FGF23 was significantly increased in vehicle-treated HMWTgFGF2 mice (Fig. 6D) and it was not reduced by BGJ398 treatment. There was also a significant increase in serum FGF23 in Vector-Control mice treated with BJG398.

### Gene expression in the TGFβ/BMP signaling pathway in knee joints of 8-month-old Vector-Control and HMWTgFGF2 mice treated with BGJ398

Total RNA extracted from knee joints of 8 month-old Vector-Control and HMWTgFGF2 male mice was used to examine whether target genes of Tgfβ/BMP signaling pathways were differentially expressed between Vector-Control and HMWTgFGF2 vehicle-treated mice. Then, the same gene expression was examined in total RNA from BJG398- or vehicle-treated Vector-Control and HMWTgFGF2 mice for comparison. The mRNA levels of 82 genes involved in the Tgfβ/BMP signaling pathway were detected using a pre-designed PrimePCR assay kit following the manufacturer's instructions. The results were analyzed using Bio-Rad CFX manager software.

**Figure 7:**
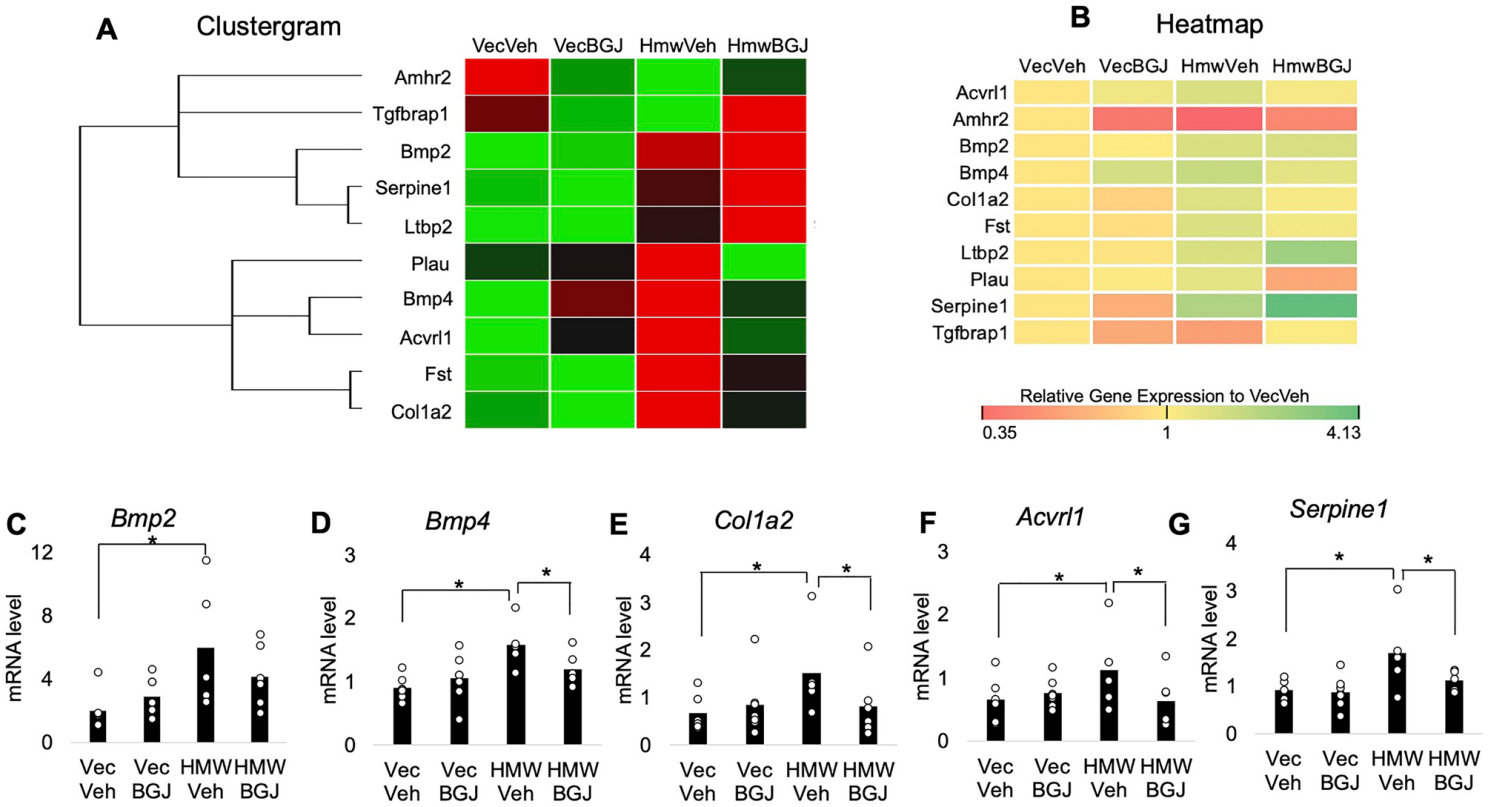
Gene expression of TGFβ/BMP signaling pathway in knee joint of 8-month-old Vector-Control and HMWTgFGF2 mice treated with BGJ398. Six and a half-month-old Vector-Control (Vec) and HMWTgFGF2 (HMW) male mice were sq injected with vehicle or BGJ398 5 days/week for 6 weeks. At the end of the experiment (8 months old) knee joints were collected and total RNA was isolated and reverse transcribed. The mRNA levels of 82 genes involved in the Tgfβ/BMP signaling pathway were detected using a pre-designed PrimePCR assay kit following the manufacturer's instructions. The results were analyzed using Bio-Rad CFX manager software. The clustergram and heatmap of the 82 genes in Tgfβ/BMP signaling pathway in Vector-Control (Vec) and HMWTgFGF (HMW) mice treated with vehicle or BGJ398 are shown in Supplemental Figs. 5 and 6. (**A**) The clustergram of the 10 target genes that were increased or decreased 1.4 fold. (**B**) Representative heatmap of the 10 differentiated genes expressed in knee samples from Vector-Control or HMWTgFGF2 mice treated with vehicle or BGJ398. HMWTgFGF2 knees display altered genes in the TGIFβt/BMP signaling pathway and rescued by BGJ398. (**C**–**G**) represent qPCR confirmation of the genes that are significantly altered in the PrimePCR array. Data are shown as mean and individual points. n = 5–7 mice/group. *p < 0.05 by two-way ANOVA.

The Clustergram and Heatmap of all 82 target genes are shown in Supplemental Figs. 5 and 6. There were 22 genes that were 1.3-fold changed in Vector-Control vehicle-treated versus HMWTgFGF2 vehicle-treated mice; and in HMWTgFGF2 vehicle-treated versus HMWTgFGF2 BGJ398-treated mice; 10 of these were 1.4-fold changed. The Clustergram Fig. 7A and Heatmap Fig. 7B represent a 1.4-fold change in 10 genes that were differentially expressed in knee samples from Vector-Control verses HMWTgFGF2 mice treated with vehicle or BGJ398. These genes were: Activin A receptor, type II-like 1(ALK1); Anti‑Mullerian hormone (Amhr2); Bone morphogenic protein 2 (Bmp2); Bone morphogenic protein 4 (Bmp4); Collagen type I alpha 2 (Col1a2); Follistatin (Fst); Latent transforming growth factor beta binding protein 2 (Ltbp2); Serine (or cysteine) peptidase inhibitor, clade E, member 1 (Serpine1); Transforming growth factor beta receptor associated protein 1(Tgfbrap1) and Plasminogen activator urokinase (Pau).

Quantitative PCR confirmed a significant increase in 7 of the 10 genes in HMWTgFGF2 vehicle-treated compared with Vector-Control vehicle-treated mice. These were: *Bmp2* (Fig. 7C), *Bmp4* (Fig. 7D), *Col1a2* (Fig. 7E), *Acvrl1* (Fig. 7F) and *Serpine1* (Fig. 7G). BGJ398 treatment reversed this effect in HMWTgFGF2 mice. The mRNAs for *Fst* and *Ltbp2* were also significantly increased in HMWTgFGF2 vehicle-treated mice, but they were not decreased by BGJ398 treatment (data not shown). The mRNA for *Amhr2* was similar in HMWTgFGF2 vehicle-treated and Vector-Control vehicle-treated mice; however, its expression was significantly increased by BGJ398 (data not shown).

### BGJ398 blocks increased expression of BMP2, BMP4 and BMP Receptor1 in knee articular cartilage of 9.5-month-old HMWTgFGF2 mice

**Figure 8:**
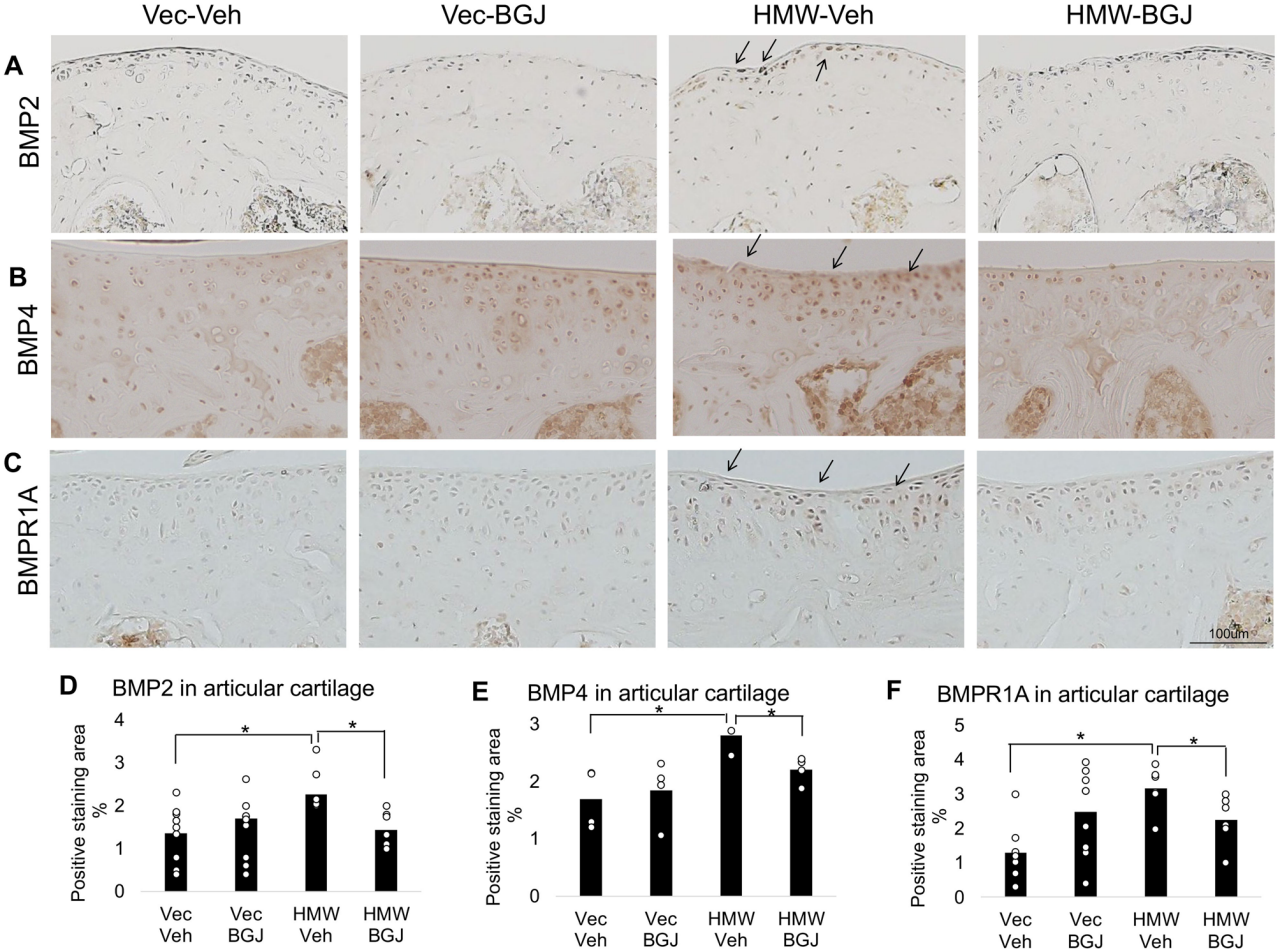
Expression of BMP2, BMP4, and BMP receptor1 in knee articular cartilage of 9.5-month-old Vector-Control and HMWTgFGF2 mice treated with BGJ398. Eight-month-old Vector-Control and HMWTgFGF2 female mice were sq injected with vehicle or BGJ398 5 days/week for 6 weeks. At the end of the experiment (9.5 months old) knee joints were collected for IHC staining. Representative immunohistochemical staining images for (**A**) BMP2, (**B**) BMP4, and (**C**) BMPR1A. Quantitative analysis of percent-positive staining area for (**D**) BMP2, n = 7–9 mice/group, (**E**) BMP4, n = 4 mice/group, and (**F**) BMPR1A, n = 7–9 mice/group, in articular cartilage. Data are shown as mean and individual points. n = 7–9 mice/group. *p < 0.05 by two-way ANOVA.

Based on the mRNA gene expression results for BMP signaling (Fig. 7) immunohistochemistry was used to examine expression of those genes on the protein level. Articular cartilage and/or subchondral bone from Vector-Control and HMWTgFGF2 mice, treated with either vehicle or BGJ398, were immune-stained for BMP2 (Fig. 8A), BMP4 (Fig. 8B) and BMPR1 (Fig. 8C). The results show marked increase in expression of all three proteins in HMWTgFGF2 vehicle-treated knee articular cartilage compared with Vector-Control vehicle-treated mice. Quantitative analysis of percent-positive staining area for BMP2 (Fig. 8D), BMP4 (Fig. 8E) and BMPR1A (Fig. 8F) reveal significant increases in HMWTgFGF2 vehicle-treated articular cartilage; that was significantly reduced by BGJ398.

### Expression of SMAD1/5/8, pSMAD6, RUNX2 and pERK1/2 in knee articular cartilage of 9.5-month-old SMAD1/5/8-pSMAD6, RUNX2 and pERK1/2 Vector-Control and HMWTgFGF2 mice treated with BGJ398

**Figure 9:**
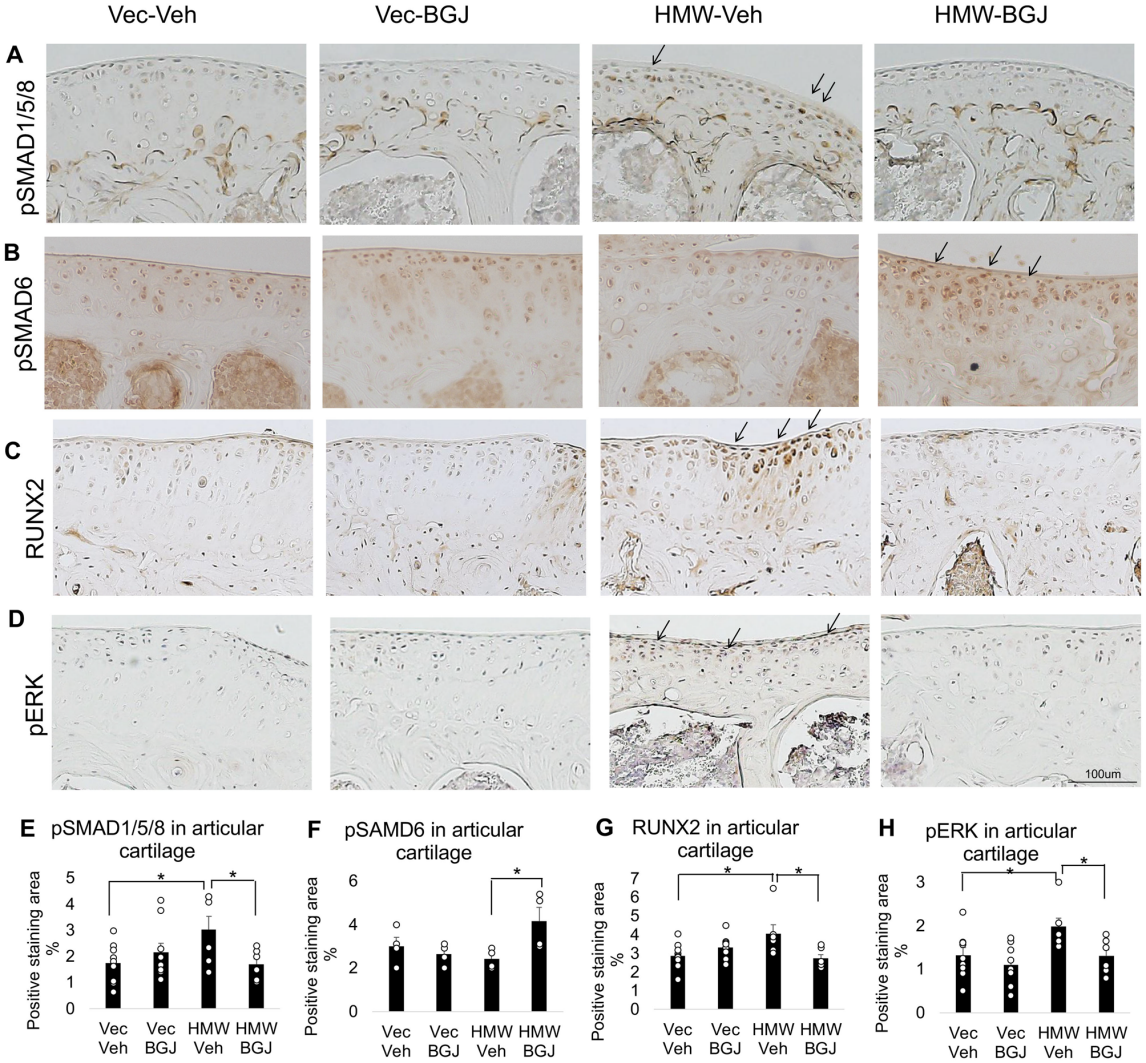
Expression of pSMAD1/5/8, pSMAD6, RUNX2 and pERK in knee articular cartilage of 9.5-month-old Vector-Control and HMWTgFGF2 mice treated with BGJ398. Eight month-old Vector-Control (Vec) and HMWTgFGF2 (HMW) female mice were sq injected with vehicle or BGJ398 5 days/week for 6 weeks. At the end of the experiment (9.5 month old) knee joints were collected for IHC staining. Representative immunohistochemical staining images for (**A**) pSMAD1/5/8, (**B**) pSMAD6, (**C**) RUNX2, and (**D**) pERK. Quantitative analysis of percent-positive staining area for (**E**) pSMAD1/5/8, (**F**) pSMAD6, (**G**) RUNX2, and (**H**) pERK in articular cartilage. Data are shown as mean and individual points. n = 7–9 mice/group for (**E**, **G**, **H**); n = 4 mice /group for (**F**). *p < 0.05 by two-way ANOVA.

BMPs can signal via canonical pathway activation of SMAD1/5/8 to increase phosphorylation of the transcription factor RUNX2, that plays a critical role in development of OA^25^. Therefore, we assessed their expression, and the inhibitory factor SMAD6, in knee articular cartilage from 9.5-month-old Vector-Control and HMWTgFGF2 female mice treated with vehicle or BGJ398. We observed a marked increase in immunohistochemical staining for pSMAD1/5/8 in HMWTgFGF2 vehicle-treated mice (Fig. 9A). By contrast staining for inhibitory pSMAD6 that can block BMPR1A signaling^27^ was reduced in HMWTgFGF2 vehicle-treated joints (Fig. 9B) and there was a marked increase in RUNX2 staining (Fig. 9C). BMPs can also signal by non-canonical pathways including activation of mitogen-activated extracellular kinases (pERK) to increase phosphorylation of RUNX2^25^; and, FGF2 signals via multiple pathways including mitogen-activated protein kinases^17^. Immunostaining (Fig. 9D) showed a marked increase in pERK expression in HMWTgFGF2 vehicle-treated knee joints compared with the Vector-Control-vehicle. Quantitative analysis for percent-positive staining area in articular cartilage for pSMAD1/5/8 (Fig. 9E), RUNX2 (Fig. 9G) and pERK (Fig. 9H) revealed significant increases in HMWTgFGF2 vehicle-treated mice compared with the Vector-Control vehicle-treated group. The increased expression for each of these proteins was completely rescued by BGJ398 treatment. Interestingly, quantitation of pSMAD6 staining (Fig. 9F) revealed a significant increase in HMWTgFGF2 BJG398-treated mice compared to HMWTgFGF2 vehicle-treated controls.

## Discussion

**Figure 10:**
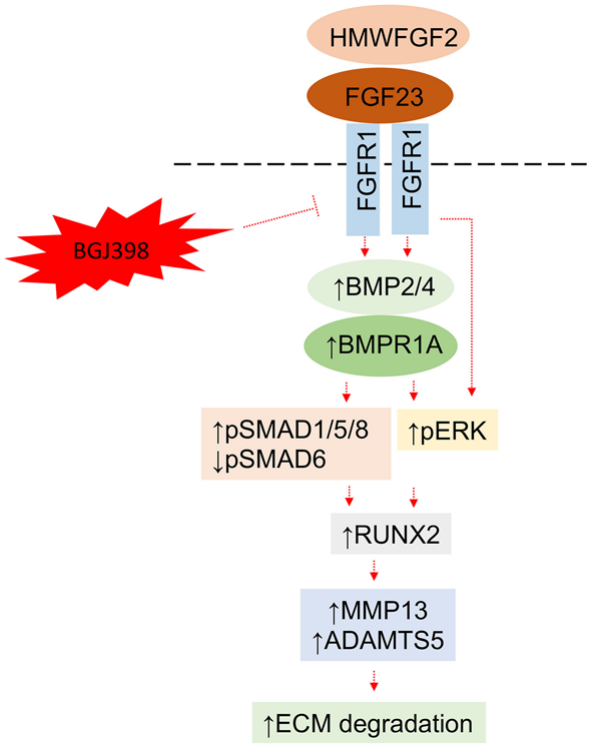
Diagrammatic proposed mechanism of signaling pathways in articular cartilage modulated by BGJ398 blocking HMWFGF2-generated OA development. HMWFGF2 increases FGF23 expression that binds to FGFR1. Activation of FGFR1 upregulates degrading enzymes MMP13 and ADAMTS5 expression via SMAD 1/5/8 and ERK regulated RUNX2 expression. FGFR inhibitor BGJ398 inhibits activation of FGFR1 and, therefore, rescues/prevents OA development in HMWTgFGF2 mice.

This study had two goals: first, to further characterize the molecular basis for OA pathogenesis by using the HWMTgFGF2 mouse model for XHL-OA studies. Second, to use the FGFR antagonist BGJ389 to determine whether long-term treatment would reverse the OA phenotype by modulating those characteristic OA regulatory molecules. The histological analysis in Figs. 1, 2 and 3 provides further evidence that the OA phenotype in HMWTgFGF2 mice is consistent with the phenotypic hallmarks for OA in Hyp mice and human XLH-OA; thereby, convincingly demonstrating that overexpression of HWMFGF2 in mice is capable of generating OA. Then, BGJ398 results provide convincing evidence that HMWFGF2 generates OA through FGF23 (Fig. 6) with activation of FGFR1 (Fig. 5). That activates downstream signaling in multiple pathways via pSMAD1/5/8-RUNX2 and pERK, ultimately upregulating the proteinases MMP13 and ADAMTS5 (Fig. 4) that degrade cartilage. Overall, these results explain how the pleotropic effects of FGF2 are mediated by the specific functions of the HMW protein isoforms for cartilage and bone homeostasis, and then XLH-degenerative osteoarthropathy (Fig. 10).

BGJ398 (infigratinib) is a small molecule inhibitor of FGFRTK signaling that was FDA approved (5/2021) for treatment of adults with metastatic cholangiocarcinoma involving *FGFR2*^28^. We previously reported that BGJ398 treatment of weanling 21-day-old HMWTgFGF2 mice prevented the development of knee joint OA. Therefore, we hypothesized that BGJ398 could partially or fully reverse severe OA in knee joints of older HMWTgFGF2 mice. The experimental results in Figs. 1, 2, 3, 4, 5, 6, 7 and 8 support that hypothesis.

The results reported here could serve as a prelude for human studies testing the efficacy of BGJ398 to treat XLH-OA. Among the well-reported phenotypic changes in generalized OA are breakdown of articular cartilage^29^, abnormal bone outgrowth or osteophytes^30^, and loss of trabeculae in subchondral bone^31^, similar findings have been reported in XLH-OA^3^. In addition, abnormal production of the matrix degenerating enzymes MMP13 and ADAMTS5^32–34^ results in destruction of articular cartilage. In the present study, radiographs of knee joints, Micro-CT structural analysis and histomorphometry of subchondral bone and articular cartilage demonstrate that there is a remarkable rescue of the subchondral bone and articular OA phenotypes by BGJ398 (Figs. 2, 3). The rescue included increased subchondral bone volume and increased articular cartilage proteoglycan, and there were significant reductions in alkaline phosphatase as well as destructive enzymes MMP13 and ADAMTS5.

The results from our previous study^26^ showed significantly reduced bone volume after 13 weeks of BGJ398 treatment in 21-day-old female Vector-Control mice. This suggests use of the FGFR inhibitor during either the growth phase of bone formation or a 13-week length of treatment (compared to 6 weeks here) could be detrimental. Those side effects could have potential translational relevance for human studies with BGJ398. It should be emphasized that we did not find any evidence of those inhibitory side effects from 6 weeks of BGJ398 treatment on the subchondral bone of 9.5-month-old female Vector-Controls in this study. There was evidence of off-target side effects on the knee joints in Vector-Control mice treated with BGJ398. Specifically, BGJ398 significantly reduced (1,25(OH)_2_D_3_) in Vector-Control mice which is most likely due to its effect to significantly increase serum FGF23 in Vector-Control mice.

Consistent with our previous report^26^, the current studies confirm a significant increase in subchondral bone FGF2 expression that was unchanged by BGJ398 treatment. Similar to our studies in weanling HMWTgFGF2 mice BGJ398 significantly reduced the observed increased FGF23 expression in articular cartilage of older HMWTgFGF2, but did not affect increased FGF23 expression in the HMWTgFGF2 subchondral bone. However, in contrast to our previous report in weanling mice, BGJ398 did not reduce increased FGF23 in serum of HMWTgFGF2 mice. This discrepancy could result from the different treatment periods, 6 weeks in older mice versus 13 weeks of treatment in weanling mice. However, even with the shorter course of treatment, BGJ398 was still efficacious, significantly reducing the HMWFGF2-mediated increase in activated FGFR1 found in knee articular cartilage from older HMWTgFGF2 mice. A similar result as was previously reported in weanling HMWTgFGF2 mice^26^. While numerous studies in both humans and mice have demonstrated a role for increased FGFR1 in articular cartilage destruction^15^, and conditional knockout of FGFR1 in knee cartilage reduced articular cartilage degeneration in mice^16^, there are no studies of the use of BGJ398 to modulate the OA phenotype in XLH subjects or Hyp mice of any age.

Other studies have demonstrated an important role for FGFR3 in normal articular cartilage homeostasis in humans and mice, based on reduced expression of FGFR3 in articular chondrocytes from humans with OA^14^ as well as development of severe OA in FGFR3 knock out mice^14^. Our studies in HMWTgFGF2 mice suggest that FGFR3 may not have a major protective role in our OA model since there were no significant FGFR3 changes observed in older HMWTgFGF2 articular cartilage by FGF receptor blockade. This observation is consistent with our reports in weanling HMWTgFGF2 mice where phosphoFGFR3 was not modulated by BGJ398, further supporting abnormal FGFR1 signaling as the primary mediator of OA in HMWTgFGF2 mice.

The PrimePCR array analysis revealed some interesting genes in the TGF-β/BMP pathway that were significantly increased in the HMWTgFGF2 vehicle-treated mice (compared with Vector-Control vehicle treatment) and then reduced by BGJ398 treatment. These results included *Col1a2* and *Serpine1*. Interestingly, increased *Col1a2* expression has previously been reported in osteoarthritic cartilage, in contrast to little or no expression in normal articular cartilage^35^. Serpine1 belongs to a family of serine proteinase inhibitors whose function, beyond inhibiting serine proteinases, is only recently emerging^36^. The role of Serpine1, that encodes for plasminogen activator inhibitor 1(PAI) in OA, is controversial since both increased and decreased expression have been reported in OA^36^. Some models suggest that osteoarthritic cartilage may lose the ability to produce protease inhibitors and that the subsequent imbalance between proteases and their inhibitors may contribute to chondropathy^37^. However, other studies indicate that protease inhibitors may be upregulated in OA^38,39^. FGF2 has been reported to increase PAI in endothelial cells^40^; however, there are no reports that either HMWFGF2 or FGF23 increases PAI.

Although phenotypic OA develops with age in XLH subjects^3,41^ the molecular mechanism(s) for osteoarthropathy observed in adult XLH subjects^41^ and the Hyp mouse homolog remains largely undefined, although FGF23/FGFR1 signaling is likely a major contributor. Other studies have shown that FGF23 drives MMP13 expression in human osteoarthritic chondrocytes in a klotho-independent manner via activation of MAPKERK^42^. Increased FGF23 and RUNX2 was reported in human articular chondrocytes compared with normal controls^43^. Moreover, down regulation of RUNX2 and FGF23 with small interfering RNA reduced MMP13 expression in human osteoarthritic chondrocytes^43^. Published evidence shows that the interplay of multiple signaling pathways regulates the fate of chondrocytes to remain within cartilage or to undergo hypertrophic differentiation^20^.

Consistent with reports showing multiple signaling pathways mediating OA, we previously reported that FGF23 neutralizing antibody partially rescued the OA phenotype in HMWTgFGF2 mice via modulation of canonical Wnt signaling^44^. However, involvement of additional signaling pathways including the TGFβ/BMP superfamily and MAPK/ERK signaling is shown by the findings of the present study that are summarized in Fig. 10. This model shows increased BMPR1, BMP2, BMP4, SMAD 1/5/8 and pERK expression consistent with our results (Figs. 8, 9), the FGF23-FGFR1 activation. Studies with cultured human OA articular chondrocyte cells show TGF-β signals predominantly through activin receptor-like kinase 1 (ALK1)/activin A receptor like type 1 (ACVRL1) SMAD1/5/8 pathways that are linked to the induction of catabolism with MMP-13^18^.

BMP signaling is important in all stages of chondrogenesis including mesenchymal condensation, chondrocyte proliferation, extracellular matrix deposition and, finally, terminal differentiation^45^. Consistent with our results here, other studies using OA mouse models have consistently demonstrated increased BMP2 and BMP4^45^, and BMP2 has been shown to upregulate MMP13 via SMAD 1/5/8 and RUNX2^39^. FGF2 can regulate expression of BMP2^46^ that increases SMAD1/5/8; and, both FGF2 and BMP2 regulate pERK which supports our model (Fig. 10) where FGF2-FGFR1-BMP crosstalk contributes to OA in HMWTgFGF2 mice. Furthermore, we previously reported that HMWFGF2 transcriptionally regulates FGF23^1^ and that FGF23/FGFR1 activates ERK, further contributing to the OA phenotype in HMWTgFGF2 mice. pERK as well as pSMAD1/5/8 signaling highlights the potential cross-talk of HMWFGF2/FGF23/FGFR/MAPK and BMPR/BMP/SMAD signaling generating OA. The results obtained here show BGJ398 can alter expression of these genes in older HMWTgFGF2 mice to modulate both pathways. Overall, our results show the multifactorial molecular profile for XLH-OA pathogenesis, that can be modelled in the HMWTgFGF2 mice and generated by overexpression of HMWFGF2. The results further reveal the pleotropic nature of FGF2 function by the HMW isoforms. The BGJ398 FGFR antagonist substantially reverses the OA phenotype, suggesting that Randomized Clinical Trials for off-label use to treat human XLH-OA could be worthwhile.

## Materials and methods

### Mice and FGFR inhibitor treatment

All animal protocols were approved by the University of Connecticut Institutional Animal Care and Use Committee (UC IACUC). Methods for generating mice expressing HMW isoforms of hFGF2 in a bone-specific manner using Col3.6-HMWFgf2 isoform-IRES-GFPsaph construct (HMWTgFGF2, HMW) and Vector-Control control mice using a Col3.6-IRES/GFPsaph construct (Vector-Control, Vec) were described previously^6^. Both male and female mice were used in this study and independent analyses for each gender was conducted.

All animal procedures were approved by the UCONN Health Institutional Animal Care and Use Committee and all experiments were performed in accordance with relevant guidelines and regulations. Additionally the studies complied with the recommendations in the ARRIVE guidelines.

The selective pan-specific FGFR inhibitor, NVP-BGJ398 (BGJ) was purchased from Chemie Tek (Indianapolis, IN). BGJ398 at 2 mg/kg body weight or vehicle only (hydrochloric acid 3.5 mM, dimethyl sulfoxide 5%) was subcutaneously injected to Vector-Control Control transgenic and HMWTgFGF2FG2 mice 5 days per week for 6 weeks. Injection was initiated at 6.5 months of age for male mice and at 8 months of age for female mice. Mice were housed by group, 4 mice per cage with ad libitum access to food or water. The male mice were sacrificed at 8 months of age and female mice were sacrificed at 9.5 months of age. Mice were euthanized by carbon dioxide for sample collection.

### In vivo faxitron X-ray

In vivo digital X-ray images in the sagittal plane of mouse knee joints were obtained at baseline and at 6 weeks post-treatment using a UltraFocus from Faxitron Bioptics LLC (Tucson, AZ) and taken under IACUC approved constant conditions.

### Serum biochemistry

Blood was collected from euthanized animals by cardiac puncture. After clotting, the blood was spun, and serum was collected. Serum calcium was measured using the StanbioTotal Calcium LiquidColor kit (StanBio Laboratory, Boerne, TX). Serum phosphate (Pi) was measured using the Stanbio Phosphorus Liqui-UV kit(StanBio Laboratory, Boerne, TX). Serum parathyroid hormone (PTH) was determined using a mouse intact PTH enzyme-linked immunosorbent assay kit (Immunotopics, Athens, OH) according to the manufacturer’s instructions. Blood Urea Nitrogen (BUN) was measured in serum using Stanbio Urea Nitrogen (BUN) Light-UV kit (StanBio Laboratory, Boerne, TX). Serum creatinine (Cr) was measured using QuantiChrom Creatinine Assay Kit (BioAssay Systems, CA). Serum 1,25(OH)_2_D_3_ was measured using 1,25-Dihydroxy Vitamin D EIA kit from Immuno Diagnostic Systems (IDS; Gaithersburg, MD). Serum intact FGF23 was measured using kits purchased from Immunotopics, Inc. (Carlsbad, CA).

### Micro–computed tomography (Micro-CT) analysis of knee joint

Knee architecture imaging was conducted using ex vivo Micro-CT, Scan resolution was 8µm^3^ in a Scanco µCT50 instrument (ScanCo Medical, Bruttisellen, Switzerland). Other scanner parameters: 55 kVp, 145 µA, 600 ms integration time, 1000 projections/180°. The epiphysial, subchondral trabecular regions in femurs were analyzed by 2-dimensional Micro-CT. We performed manual slice-by-slice contouring of the epiphyseal trabecular bone of both the distal femur and proximal tibia, excluding all cortex. Standard trabecular analysis was performed in Scanco evaluation software using a threshold of 315 per mille. Bone volume/tissue volume (BV/TV), trabecular thickness (Tb.Th), trabecular number (Tb.N), and trabecular spacing (Tb.Sp) were calculated and analyzed.

### Histologic analysis of knee joint

Knee joints were dissected and fixed in 10% neutral-buffered formalin for 48 h, then decalcified with 14% EDTA solution at 4 °C for 7 days. Samples were processed for paraffin embedding in a frontal orientation and 7-μm sections were obtained. Following de-paraffinization and rehydration of the sections, Safranin-O staining of glycosaminoglycans was performed using 0.1% aqueous Safranin-O and counterstained with Weigert’s iron hematoxylin and 0.02% aqueous Fast Green. Alkaline phosphatase staining was performed by incubating tissue with 100 mM Tris–HCl buffer (pH, 8.2) for 10 min, followed by incubation with Vector-Control Blue Alkaline Phosphatase Substrate (Vector-Control Laboratories Inc, Burlingame, CA) for 30 min. The severity of cartilage destruction was assessed histologically on all articular surfaces within each representative section from each knee using Safranin-O stained sections. The bone shape was different between Vec and HMW mice. Knee joints were sectioned frontally, and the sharp triangle shape meniscus on both sides were used as land mark for section used for the histology assessment. We did serial sectioning and could only collect 20 sections per knee. Section number 10 for each knee was used for scoring. The assessment was performed by an experienced researcher and the slides were blinded prior to evaluation. The following recommend scoring system from Glasson et al.^26^ was used: 0 = normal; 0.5 = loss of proteoglycan without structural changes, 1 = small fibrillation without cartilage loss, 2 = vertical clefts down to layer directly beneath superficial layer/some loss of surface lamina, 3 = vertical clefts/erosion down to calcified cartilage layer which extends < 25% of articular surface, 4 = vertical clefts/erosion down to calcified cartilage layer which extends 25–50% of articular surface, 5 = vertical clefts/erosion down to calcified cartilage layer which extends 50–75% of articular surface, 6 = vertical clefts/erosion down to calcified cartilage layer which extends > 75% of articular surface.

### Immunohistochemistry

Paraffin-embedded 7-μm sections of knee joints were used for immunohistochemical staining. Sections were de-paraffinized and rehydrated, endogenous peroxidase activity was blocked by incubating sections with 3% hydrogen peroxide in water for 15 min. Then slides underwent antigen retrieval by incubation at 60 °C with 10 mM sodium citrate buffer for overnight. The tissue was blocked with 10% serum for 30 min at room temperature, then the slides were incubated with primary antibodies in blocking buffer for 1 h. The following primary antibodies were used: rabbit anit-MMP13 (1:100, Abcam, ab39012), rabbit anti-ADAMTS5 (1:100, Abcam, ab182795), rabbit anti-FGF2 (1:50, Santa Cruz, sc-79), rabbit anti-pFGFR1 (1:50, Abnova, PAB0471), rat anti-FGF23 (1:50, R&D SYSTEMS, MAB26291), rabbit anti-BMP2 (1:100, Abcam, ab14933), rabbit anti-BMP4 (1:200, NovusBio, NBP2-49477), rabbit anti-BMPR1A (1:200, Abcam, ab264043), rabbit anti-pSMAD1/5/8 (1:50, Millipore Sigma, AB3848-I), rabbit anti-pSMAD6 (1:50, Affinity Biosciences, AF3769), rabbit anti-phosphop44/42 MAPK (1:400, Cell Signaling, 4376S), and mouse anti-RUNX2 (10 μg/ml, MBL, D130-3, M.O.M kit was used). Slides were then washed with phosphate-buffered saline and incubated with the appropriate biotinylated secondary antibody at room temperature for 1 h. Then slides were washed and developed with DAB Peroxidase Substrate kit (Vector-Control Laboratories, Burlingame, CA), and counterstained with Harris hematoxylin. The percentage of positive staining area was determined using the OsteoMeasure image analysis system (R & M Biometrics, Nashville, TN) equipped with a Nikon E400 microscope (Nikon Inc., Melville, NY).

### RNA isolation and gene analysis

Total RNA was extracted from knee joints using Trizol reagent (Invitrogen Life Technologies, Carlsbad, CA, USA). Then RNA was reverse-transcribed using the RNA to cDNA EcoDry™ Premix (Oligo dT). The mRNA levels of 82 genes involved in the Tgfβ/BMP signaling pathway were detected using a pre-designed PrimePCR assay kit following the manufacturer's instructions (BIO-RAD Laboratories Inc., Hercules, CA, USA). The PrimePCR Array results were analyzed using Bio-Rad CFX manager software. The clustergram of target genes was generated using the Gene Study software from Bio-Rad. The heatmap was generated in MSExcel. The genes in the PrimePCR array displaying a 1.4-fold difference in HMW-Vec vs. Vec-Veh and HMW-BGJ398 vs. HMW-Veh were confirmed using real-time quantitative RT-PCR (qPCR) analysis. qPCR was carried out using the iTaq™ Universal SYBR^®^ Green Supermix on a MyiQ™ instrument (BIO-RAD Laboratories Inc., Hercules, CA, USA). Gapdh was used as an internal reference for each sample. mRNA was normalized to the Gapdh mRNA level and expressed as the fold-change relative to the first sample for each experimental group. Relative mRNA expression was calculated using a formula reported previously^26^. The primers for the genes of interest are listed in Table 1.

**Table 1:** Primers used for RT-qPCR.

Gene	Forward	Reverse
*Gapdh*	5′CAGTGCCAGCCTCGTCCCGTAGA-3′	5′-CTGCAAATGGCAGCCCTGGTGAC-3′
*Acvrl1*	5′-GGGCCTTTTGATGCTGTCG-3′	5′-TGGCAGAATGGTCTCTTGCAG-3′
*Amhr2*	5′-CAGCATGACCATATCGTTCGC-3′	5′-GGAGCCCTTAGGGTACAGTTC-3′
*Bmp2*	5′-AGCGTCAAGCCAAACACAAACAG-3′	5′-GGTTAGTGGAGTTCAGGTGGTCAG-3′
*Bmp4*	5′-ATTCCTGGTAACCGAATGCTG-3′	5′-CCGGTCTCAGGTATCAAACTAGC-3′
*Col1a2*	5′-AAGGGTGCTACTGGACTCCC-3′	5′-TGTTACCGGATTCTCCTTTGG-3′
*Fst*	5′-CCCCAGACTGTTCCAACATCA-3′	5′-CACATTCGTTGCGGTAGGTT-3′
*Ltbp2*	5′-GCTCACCGGGAGAAATGTCTG-3′	5′-CAGGTTTGATACAGTGGTTGGT-3′
*Serpine1*	5′-GTGAATGCCCTCTACTTCAGTG-3′	5′-GCTGCCATCAGACTTGTGGAA-3′
*Tgfbrap1*	5′-TGCAGATTGTCAAGGAAGTGTC-3′	5′-AGGCCAGGCACAAGAAATAGC-3′
*Plau*	5′-GCGCCTTGGTGGTGAAAAAC-3′	5′-TTGTAGGACACGCATACACCT-3′

### Statistical analysis

Sample size and power was calculated based on the epiphysis BV/TV of HMW-Vec and HMW-BGJ weanling mice in our previous publication^26^. For this condition, a mean difference of 3 and standard deviation of 2 for each group, achieve 80% power to detect this difference with 7 mice per group at a 0.05 significance level sided at 2. Normality test was conducted using a Shapir-Wilk’s test (p > 0.05) and a visual inspection of histograms, normal Q-Q plots and box plots using SPSS software (IBM Corp., Armonk, NY). SPSS software was used for statistical analysis using (two way) ANOVA followed by least significant difference (LSD) for post hoc multiple comparisons. Differences were considered significant at p < 0.05. Data are shown as mean and individual points.

## Supplementary Information

Supplementary Figures.

## Data Availability

The datasets generated during and/or analyzed during the current study are available from the corresponding author on reasonable request.

## References

[1] Econs MJ, Francis F (1997). Positional cloning of the PEX gene: New insights into the pathophysiology of X-linked hypophosphatemic rickets. Am. J Physiol Renal Physiol..

[2] Hardy DC, Murphy WA, Siegel BA, Reid IR, Whyte MP (1989). X-linked hypophosphatemia in adults: Prevalence of skeletal radiographic and scintigraphic features. Radiology.

[3] Reíd IR (1989). X-linked hypophosphatemia: A clinical, biochemical, and histopathologic assessment of morbidity in adults. Medicine.

[4] Liang G, Katz LD, Insogna KL, Carpenter TO, Macica CM (2009). Survey of the enthesopathy of X-linked hypophosphatemia and its characterization in Hyp mice. Calcif. Tissue Int..

[5] Liang G, VanHouten J, Macica CM (2011). An atypical degenerative osteoarthropathy in Hyp mice is characterized by a loss in the mineralized zone of articular cartilage. Calcif. Tissue Int..

[6] Xiao L (2010). Nuclear isoforms of fibroblast growth factor 2 are novel inducers of hypophosphatemia via modulation of FGF23 and KLOTHO. J. Biol. Chem..

[7] Meo Burt P, Xiao L, Dealy C, Fisher MC, Hurley MM (2016). FGF2 high molecular weight isoforms contribute to osteoarthropathy in male mice. Endocrinology.

[8] Orito K, Koshino T, Saito T (2003). Fibroblast growth factor 2 in synovial fluid from an osteoarthritic knee with cartilage regeneration. J. Orthop. Sci..

[9] Im HJ (2009). Basic fibroblast growth factor accelerates matrix degradation via a neuro-endocrine pathway in human adult articular chondrocytes. J. Cell Physiol..

[10] Chia SL (2009). Fibroblast growth factor 2 is an intrinsic chondroprotective agent that suppresses ADAMTS-5 and delays cartilage degradation in murine osteoarthritis. Arthritis Rheumatol..

[11] Meo Burt P, Xiao L, Doetschman T, Hurley MM (2019). Ablation of low-molecular-weight FGF2 isoform accelerates murine osteoarthritis while loss of high-molecular-weight FGF2 isoforms offers protection. J. Cell Physiol..

[12] Okada-Ban M, Thiery JP, Jouanneau J (2000). Fibroblast growth factor-2. Int. J. Biochem. Cell Biol..

[13] Azhar M (2009). Gene targeted ablation of high molecular weight fibroblast growth factor-2. Dev. Dynam..

[14] Ellman MB (2013). Fibroblast growth factor control of cartilage homeostasis. J. Cell Biochem..

[15] Yan D (2011). Fibroblast growth factor receptor 1 is principally responsible for fibroblast growth factor 2-induced catabolic activities in human articular chondrocytes. Arthritis Res. Ther..

[16] Weng T (2012). Genetic inhibition of fibroblast growth factor receptor 1 in knee cartilage attenuates the degeneration of articular cartilage in adult mice. Arthritis. Rheum..

[17] Muddasani P, Norman JC, Ellman M, van Wijnen AJ, Im HJ (2007). Basic fibroblast growth factor activates the MAPK and NFκB pathways that converge on Elk-1 to control production of matrix metalloproteinase-13 by human adult articular chondrocytes. J. Biol Chem..

[18] Boehme KA, Rolauffs B (2018). Onset and progression of human osteoarthritis—Can growth factors, inflammatory cytokines, or differential miRNA expression concomitantly induce proliferation, ECM degradation and inflammation in articular cartilage?. Int. J. Mol Sci..

[19] Li X (2012). Species-specific biological effects of FGF-2 in articular cartilage: Implication for distinct roles within the FGF receptor family. J. Cell Biochem..

[20] Zhong L, Huang X, Karperien M, Post JN (2015). The regulatory role of signaling crosstalk in hypertrophy of MSCs and human articular chondrocytes. Int. J Mol Sci..

[21] Van der Kraan PM, Blaney Davidson EN, van den Berg WB (2010). Bone morphogenetic proteins and articular cartilage: To serve and protect or a wolf in sheep clothing’s?. Osteoarthr. Cartil..

[22] Fukui N, Zhu Y, Maloney WJ, Clohisy J, Sandell LJ (2003). Stimulation of BMP-2 expression by pro-inflammatory cytokines IL-1 and TNF-alpha in normal and osteoarthritic chondrocytes. J. Bone J. Surg. Am..

[23] Nakase T (2003). Localization of bone morphogenetic protein-2 in human osteoarthritic cartilage and osteophyte. Osteoarthr. Cartil..

[24] Papathanasiou I, Malizos KN, Tsezou A (2012). Bone morphogenetic protein-2-induced Wnt/beta-catenin signaling pathway activation through enhanced low-density-lipoprotein receptor-related protein 5 catabolic activity contributes to hypertrophy in osteoarthritic chondrocytes. Arthritis. Res. Ther..

[25] Schmal H (2012). Expression of BMP-receptor type 1A correlates with progress of osteoarthritis in human knee joints with focal cartilage lesions. Cytotherapy.

[26] Xiao L, Williams D, Hurley MM (2020). Inhibition of FGFR signaling partially rescues osteoarthritis in mice overexpressing high-molecular-weight FGF2 isoforms. Endocrinology.

[27] Imamura T (1997). Smad6 inhibits signalling by the TGF-β superfamily. Nature.

[28] Botrus G, Raman P, Oliver T, Bekaii-Saab T (2021). Infigratinib (BGJ398): An investigational agent for the treatment of FGFR-altered intrahepatic cholangiocarcinoma. Expert. Opin. Investig. Drugs..

[29] Karvone RL (1994). Factors affecting articular cartilage thickness in osteoarthritis and aging. J. Rheumatol..

[30] Altman R (1986). Development of criteria for the classification and reporting of osteoarthritis: Classification of osteoarthritis of the knee. Arthritis Rheum..

[31] Hayami T (2006). Characterization of articular cartilage and subchondral bone changes in the rat anterior cruciate ligament transection and menisectomized models of osteoarthritis. Bone.

[32] Mitchell PG (1996). Cloning, expression, and type II collagenolytic activity of matrix metalloproteinase-13 from human osteoarthritic cartilage. J. Clin. Invest..

[33] Glasson SS (2005). Deletion of active ADAMTS5 prevents cartilage degradation in a murine model of osteoarthritis. Nature.

[34] Stanton H (2005). ADAMTS5 is the major aggrecanase in mouse cartilage in vivo and in vitro. Nature.

[35] Hermansson M (2004). Proteomic analysis of articular cartilage shows increased type II collagen synthesis in osteoarthritis and expression of inhibin beta A (activin A), a regulatory molecule for chondrocytes. J. Biol. Chem..

[36] Wilkinson DJ (2021). Serpins in cartilage and osteoarthritis: What do we know?. Biochem. Soc. Trans..

[37] Franses RE, McWilliams DF, Mapp PI, Walsh DA (2010). Osteochondral angiogenesis and increased protease inhibitor expression in OA. Osteoarthritis Cartilage.

[38] Kevorkian L (2004). Expression profiling of metalloproteinases and their inhibitors in cartilage. Arthritis Rheum..

[39] Treadwell BV, Pavia M, Towle CA, Cooley VJ, Mankin HJ (1991). Cartilage synthesizes the serine protease inhibitor PAI-1: support for the involvement of serine proteases in cartilage remodeling. J. Orthop. Res..

[40] Kaneko T (2002). Induction of plasminogen activator inhibitor-1 in endothelial cells by basic fibroblast growth factor and its modulation by fibric acid. Arterioscler. Thromb. Vasc. Biol..

[41] Steele A (2020). Osteoarthritis, osteophytes, and enthesophytes affect biomechanical function in adults with X-linked hypophosphatemia. J. Clin. Endocrinol. Metab..

[42] Bianchi A (2016). Fibroblast Growth Factor 23 drives MMP13 expression in human osteoarthritic chondrocytes in a klotho-independent manner. Osteoarthritis Cartilage.

[43] Orfanidou T, Iliopoulos D, Malizos KN, Tsezou A (2009). Involvement of SOX-9 and FGF-23 in RUNX-2 regulation in osteoarthritic chondrocytes. J. Cell. Mol. Med..

[44] Meo Burt P, Xiao L, Hurley MM (2018). FGF23 regulates Wnt/β-catenin signaling-mediated osteoarthritis in mice overexpressing high molecular weight FGF2. Endocrinology.

[45] van Kraan PM, Blaney Davidson EN, van Berg WB (2010). Bone morphogenetic proteins and articular cartilage. To serve and protect or a wolf in sheep clothing’s?. Osteoarthritis Cartilage.

[46] Fakhry A (2005). Effects of FGF-2/-9 in calvarial bone cell cultures: Differentiation stage dependent mitogenic effect, inverse regulation of BMP-2 and noggin, and enhancement of osteogenic potential. Bone.

