# Supplementary material for: FGF receptor inhibitor BGJ398 partially rescues osteoarthritis-like phenotype in older high molecular weight FGF2 transgenic mice via multiple mechanisms
Source: Sci Rep. 2022 Sep 24;12:15968. doi: 10.1038/s41598-022-20269-6 (PMC9509331; doi:10.1038/s41598-022-20269-6)
Supplement: Supplementary file 1 — Supplementary Figures. [file 41598_2022_20269_MOESM1_ESM.pdf]

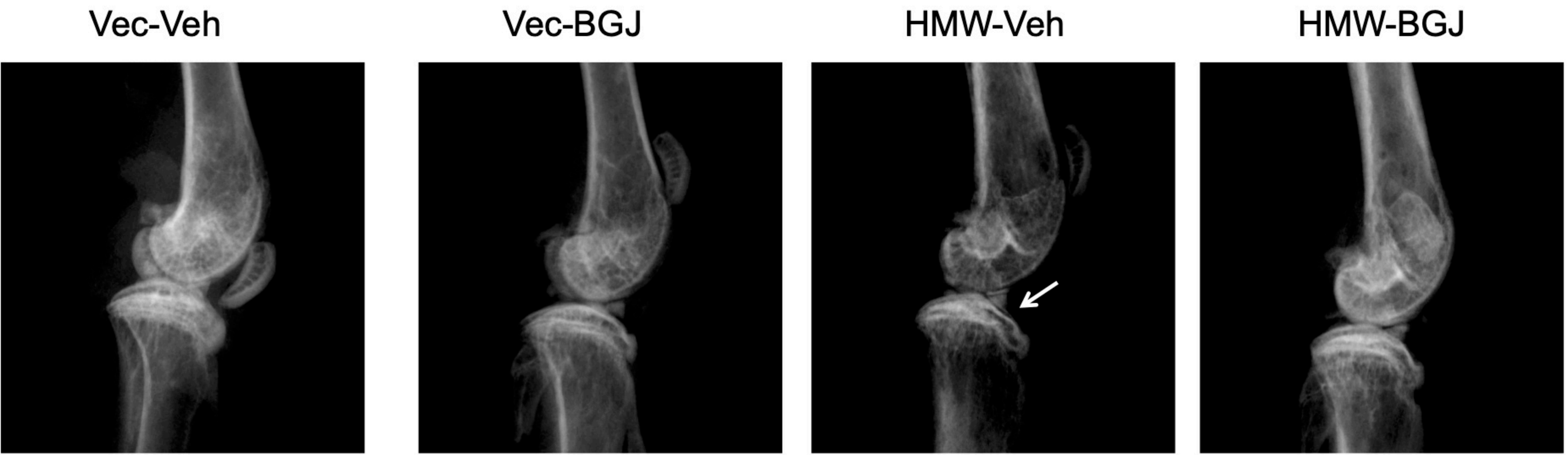

Supplemental Figure 1. X-rays of knees of 8 months-old Vector and HMWTg male mice treated with vehicle or BGJ398. These images were taken at UConn Health.

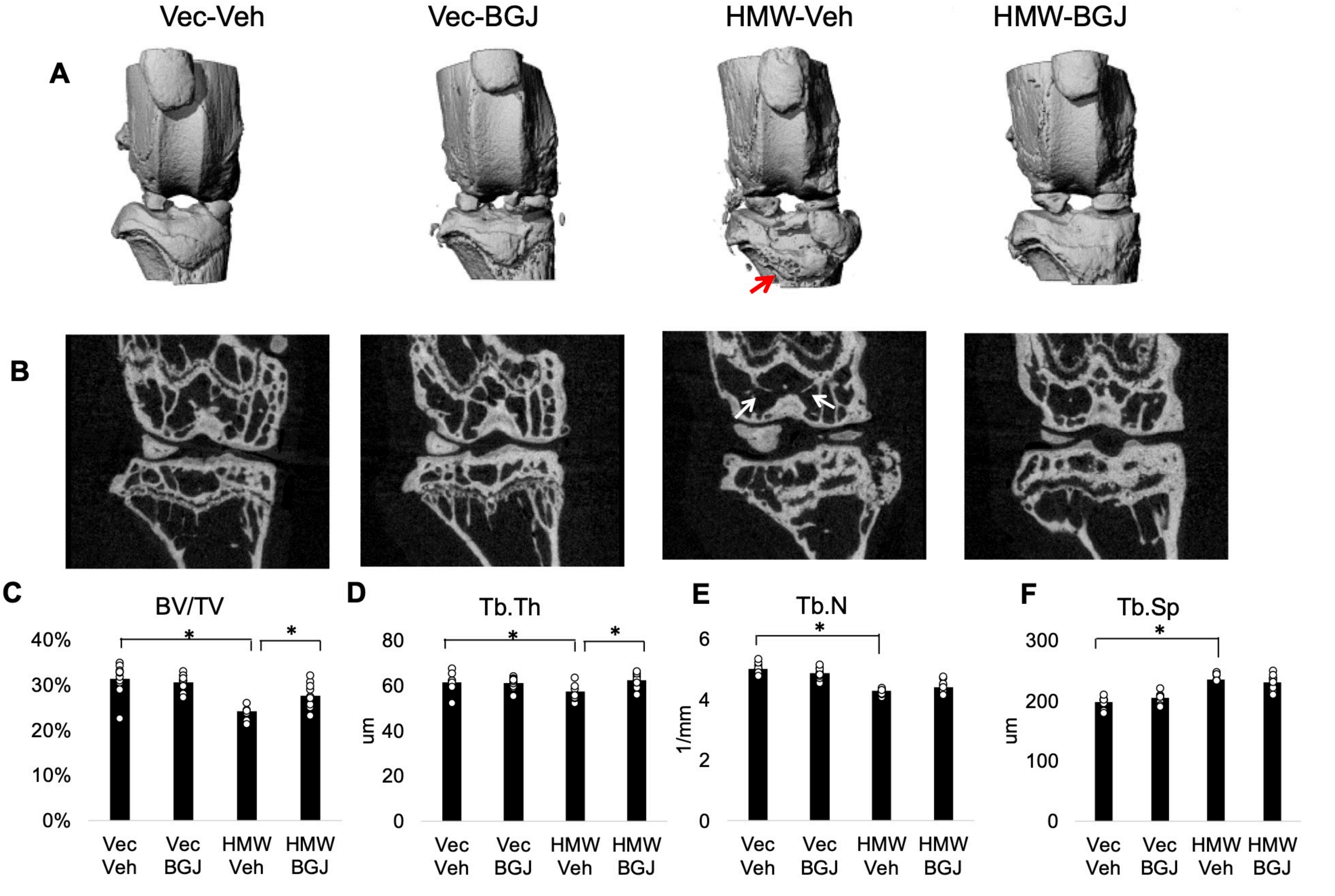

Supplemental Figure 2. Micro-CT of knees of 8 months-old Vector and HMWTg male mice treated with vehicle or BGJ398. These images were taken at UConn Health.

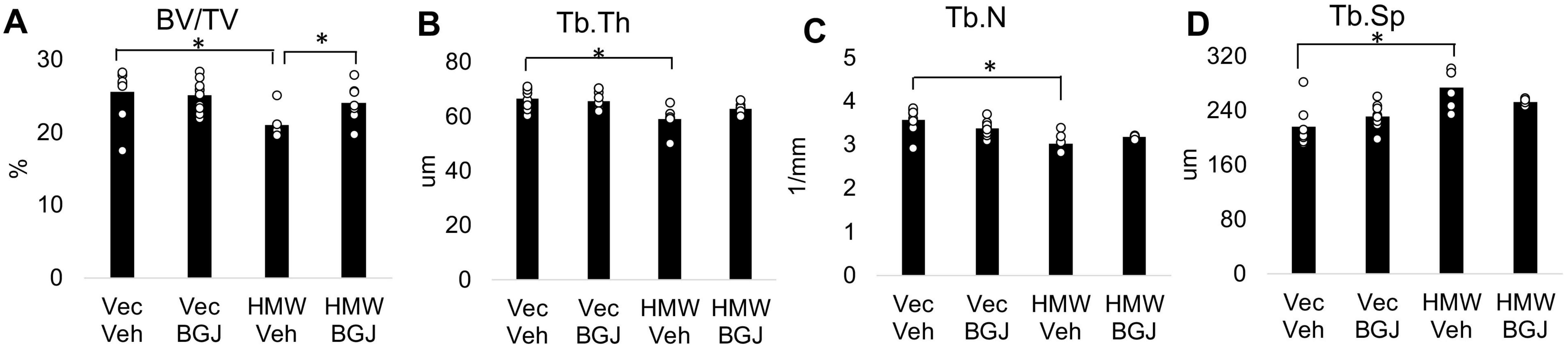

Supplemental Figure 3. Histomorphometry analysis of the femoral subchondral bone from 9.5-month-old Vector and HMWTgFGF2 female mice treated with vehicle or BGJ398.

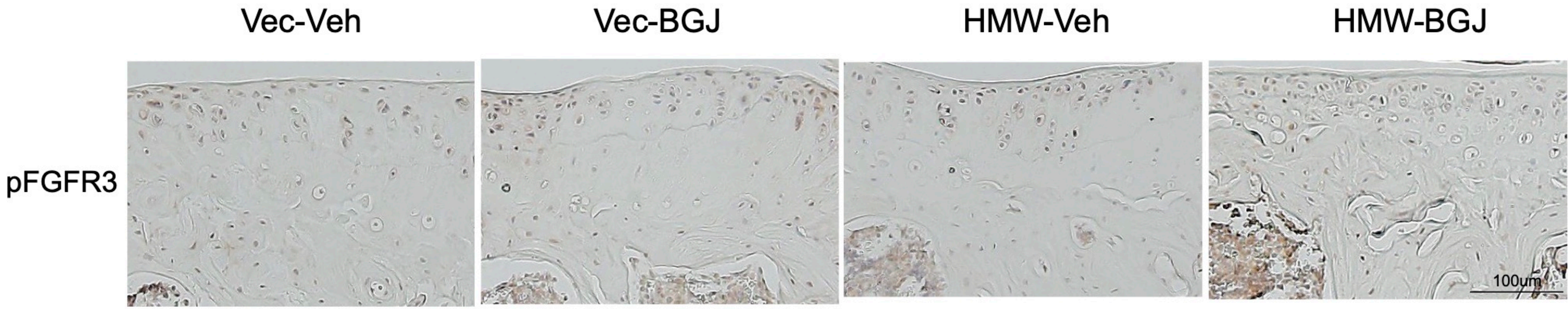

Supplemental Figure 4. IHC staining shows no difference in pFGFR3 expression among groups. These images were taken at UConn Health.

Clustergram

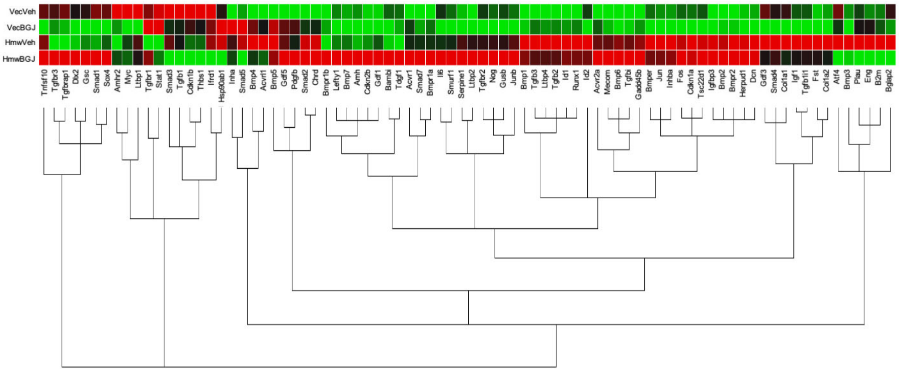

Supplemental Figure 5. Clustergram of genes of TGFβ/BMP signaling pathway in knee joint of 8 months-old Vector and HMWTg mice treated with vehicle or BGJ398.

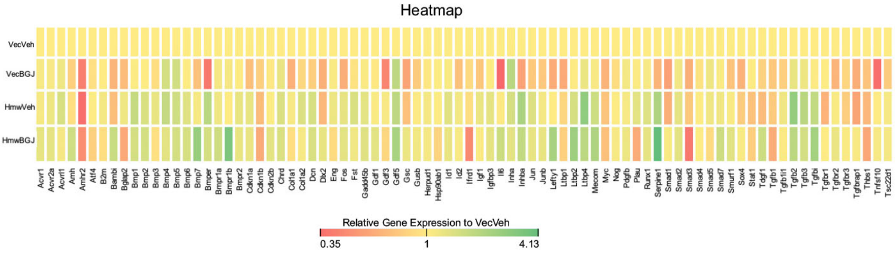

Supplemental Figure 6. Heatmap of genes of TGFβ/BMP signaling pathway in knee joint of 8 months-old Vector and HMWTg mice treated with vehicle or BGJ398.

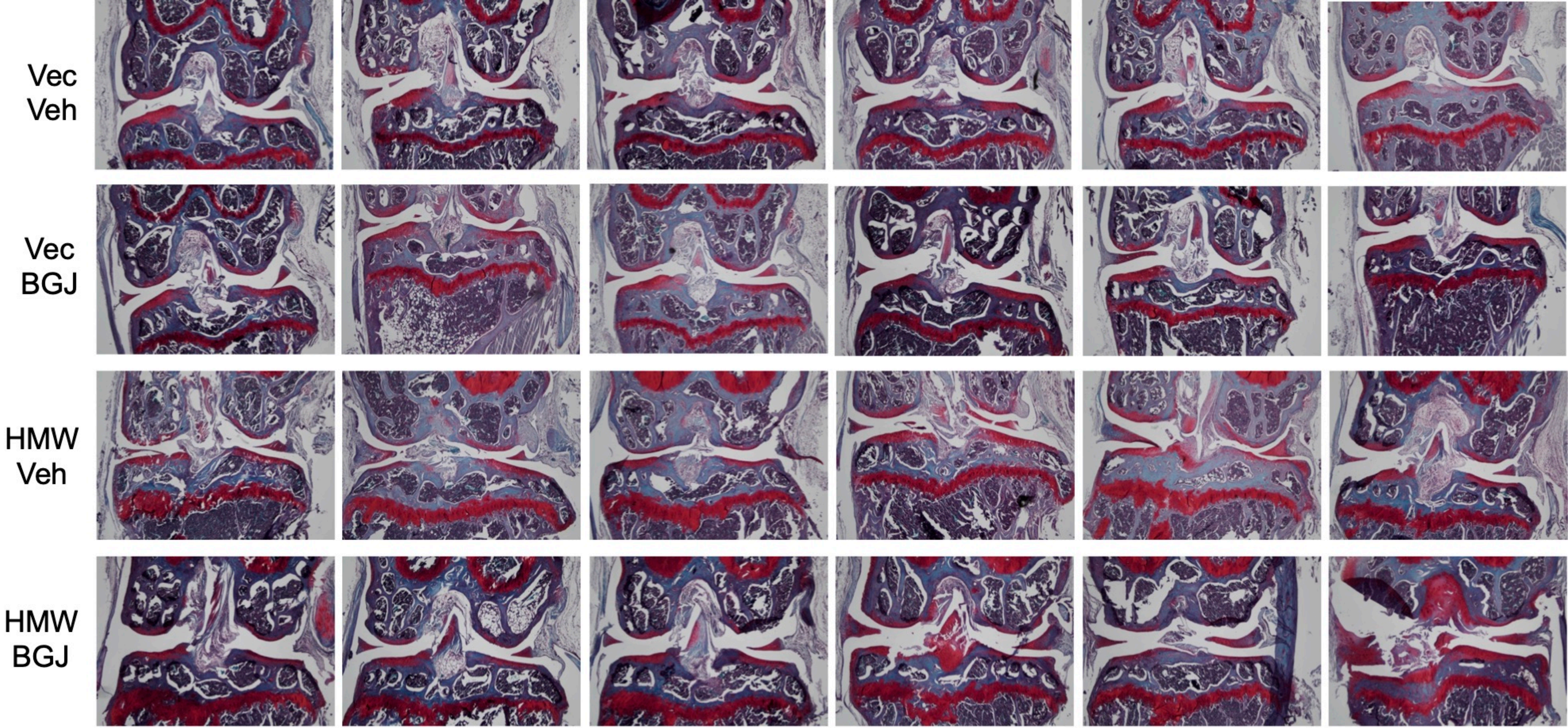

Supplemental Figure 7. Safranin-O-stained images of knee joint that are used for OA scoring. These images were taken at UConn Health.
